# Boosting Hydrogen Evolution Reaction Activity of Amorphous Molybdenum Sulfide Under High Currents Via Preferential Electron Filling Induced by Tungsten Doping

**DOI:** 10.1002/advs.202202445

**Published:** 2022-07-25

**Authors:** Dai Zhang, Feilong Wang, Wenqi Zhao, Minghui Cui, Xueliang Fan, Rongqing Liang, Qiongrong Ou, Shuyu Zhang

**Affiliations:** ^1^ Institute of Future Lighting Academy for Engineering and Technology Fudan University Shanghai 200433 P.R. China; ^2^ Institute for Electric Light Sources School of Information Science and Technology Fudan University Shanghai 200433 P.R. China; ^3^ Department of Chemistry Shanghai Key Laboratory of Molecular Catalysis and Innovative Materials Laboratory of Advanced Materials and Collaborative Innovation Center of Chemistry for Energy Materials Fudan University Shanghai 200433 P.R. China

**Keywords:** amorphous molybdenum tungsten sulfide, amorphous transition‐metal sulfides, density functional theory, high current density, hydrogen evolution reaction, plasma treatment

## Abstract

The lack of highly efficient, durable, and cost‐effective electrocatalysts for the hydrogen evolution reaction (HER) working at high current densities poses a significant challenge for the large‐scale implementation of hydrogen production from renewable energy. Herein, amorphous molybdenum tungsten sulfide/nitrogen‐doped reduced graphene oxide nanocomposites (a‐MoWS_x_/N‐RGO) are synthesized by plasma treatment for use as high‐performance HER catalysts. By adjusting the plasma treatment duration and chemical composition, an optimal a‐MoWS_x_/N‐RGO catalyst is obtained, which exhibits a low overpotential of 348 mV at a current density of 1000 mA cm^−2^ and almost no decay after 24 h of working at this current density, outperforming commercial platinum/carbon (Pt/C) and previously reported heteroatom‐doped MoS_2_‐based catalysts. Based on density functional theory (DFT) calculations, it is found that with a reasonable tungsten doping level, the catalytic active site (2S^2 −^ ) shows excellent catalytic performance working at high current densities because extra electrons preferentially fill at 2S^2 −^ . The introduction of tungsten tends to lower the electronic structure energy, resulting in a closer‐to‐zero positive $\Delta G_{H^{*}}$. Excessive tungsten introduction, however, can lead to structural damage and a worse HER performance under high current densities. The work provides a route towards rationally designing high‐performance catalysts for the HER at industrial‐level currents using earth‐abundant elements.

## Graphical abstract

This work describes a facile method for synthesizing amorphous molybdenum tungsten sulfide/nitrogen‐doped reduced graphene oxide nanocomposites toward efficient high‐current‐density hydrogen evolution reactions (HER). Density functional theory (DFT) calculations prove that 2S^2 −^ can be used to maintain high activity with electron injection and the introduction of tungsten tends to induce a closer‐to‐zero positive $\Delta G_{H^{*}}$ under high current densities.

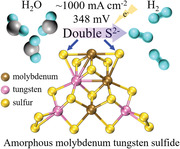

## 1 Introduction

The extensive consumption of fossil fuels has triggered ever‐increasing worldwide concerns over the energy crisis and environmental pollution. Hydrogen has attracted considerable interest as an ideal energy carrier with its high energy density, environmental friendliness, and renewability.^[^ ^1^, ^2^ ^]^ Electrochemical water splitting driven by electricity generated from renewable energy (e.g., wind and solar) is a significant, carbon‐neutral approach for producing green hydrogen.^[^ ^3^ ^]^ Typically, platinum and platinum‐based materials are used widely as cathodic HER catalysts to overcome sluggish kinetics in water electrolysis.^[^ ^4^ ^]^ The scarce reserve and prohibitive price of platinum‐based catalysts, however, has severely hindered the large‐scale deployment of water electrolysis technology.^[^ ^5^ ^]^ Thus, a great deal of effort has been devoted to developing platinum‐group‐metal‐free (PGM‐free) HER catalysts, such as transition‐metal carbides,^[^ ^6^, ^7^ ^]^ nitrides,^[^ ^8^, ^9^ ^]^ sulfides,^[^ ^10^, ^11^, ^12^, ^13^ ^]^ and phosphides.^[^ ^14^, ^15^, ^16^ ^]^ Among these PGM‐free catalysts, transition‐metal disulfides (TMS), such as MoS_2_ and WS_2_, are generally considered to be promising candidates because of their low cost and high catalytic activity.^[^ ^17^ ^]^ Both theoretical and experimental results have indicated that, in the crystalline state, only the edge sulfur sites of MoS_2_ and WS_2_ are catalytically active for the HER, and the vast amount of sulfur atoms in the basal plane are inert,^[^ ^18^, ^19^, ^20^ ^]^ which has led to much lower HER activity for TMS than that of commercial platinum/carbon (Pt/C). Conversely, amorphous materials have been proven to be better catalysts for water electrolysis than their crystalline counterparts because of the abundant active sites in amorphous structures.^[^ ^21^, ^22^, ^23^, ^24^ ^]^ For practical uses, the performance of electrocatalysts at high current densities is critical. For example, viable catalysts for commercial electrolyzers should operate stably at high current densities of more than 500 mA cm^−2^.^[^ ^25^ ^]^ Amorphous catalysts usually exhibit inferior stability, especially under high‐current‐density conditions.^[^ ^17^ ^]^ Therefore, it is highly desirable and challenging to synthesize amorphous transition‐metal sulfides (a‐TMS) that possess a low overpotential (*η*) and excellent stability at large current densities.

Plasma technology is an effective tool for the fabrication and modification of low‐dimensional nanomaterials, and the rich highly reactive species present in plasma can substantially accelerate chemical reactions.^[^ ^26^ ^]^ In particular, plasma can be employed to synthesize content‐controllable amorphous materials under a nonthermal equilibrium state because of its intrinsically high reactivity at room temperature and non‐heating effect.^[^ ^27^, ^28^ ^]^ Inspired by this idea, we hypothesized that a high‐performance amorphous alloyed TMS catalyst can be fabricated using plasma technology. In this study, we synthesized amorphous molybdenum tungsten sulfide/nitrogen‐doped reduced graphene oxide (a‐MoWS_x_/N‐RGO) nanocomposites for high‐current‐density HER using argon and ammonia (Ar + NH_3_) plasma treatment, investigated their structural and HER characteristics, and explored the mechanisms by which these catalysts can work efficiently at high current densities. Although MoS_2_‐based catalysts have been studied extensively, previous reports have focused on the performance of HER under small current densities (<100 mA cm^−2^). To the best of our knowledge, few studies have explored MoS_2_‐based materials as high‐performance catalysts toward high‐current‐density HER. In this work, we developed this earth‐abundant material into efficient HER catalysts under large current densities. The reported a‐MoWS_x_/RGO catalyst delivered a current density of 1000 mA cm^−2^ at a low overpotential of 348 mV, and its performance showed no decay after continuous 24 h electrolysis at this current density. The mechanisms we proposed to interpret its excellent performance at high current densities, which are preferential electron filling at 2S^2 −^ and the lowering of electronic structure energy induced by tungsten doping, provide guidance for the rational design of high‐performance HER catalysts under industrial‐level currents.

## 2 Results and Discussion

### 2.1 Structural Characteristics of a‐MoWS_x_/N‐RGO

**Figure 1:**
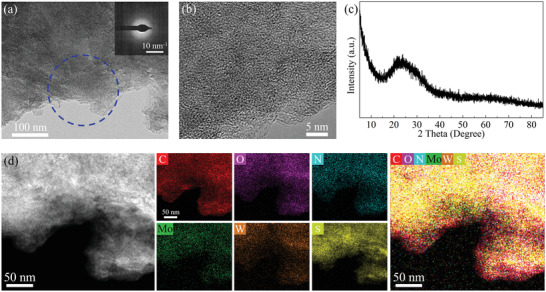
Structural and elemental characterization of the a‐MoWS_x_/N‐RGO@1:1–10 min nanocomposite. a) Medium‐magnification TEM image of a‐MoWS_x_/N‐RGO (inset shows the SAED pattern of the area marked by the blue dotted circle). b) High‐resolution TEM image of a‐MoWS_x_/N‐RGO. c) XRD pattern of a‐MoWS_x_/N‐RGO. d) HAADF‐STEM image and corresponding elemental mapping of C, O, N, Mo, W, and S and their overlay.

We completed the fabrication of a‐MoWS_x_/N‐RGO using a custom‐made radio frequency inductively coupled plasma system. The details for the preparation procedure are given in the experimental section of the Supporting Information. We named the as‐fabricated samples a‐MoWS_x_/N‐RGO@X:X‐YY (where X:X denotes the ratio of (NH_4_)_2_MoS_4_ to (NH_4_)_2_WS_4_ and YY denotes the plasma treatment duration). The morphology and structure of the a‐MoWS_x_/N‐RGO@1:1–10 min nanocomposite were first characterized. The medium‐magnification transmission electron microscopy (TEM) image (**Figure** **1**a) shows that a‐MoWS_x_/N‐RGO exhibited a lamellar morphology, which is typical for 2D‐TMS/RGO nanocomposites.^[^ ^29^, ^30^ ^]^ The inset in Figure 1a shows the selected‐area electron diffraction (SAED) pattern for the area marked with a dotted circle. Diffraction spots were not identified, which proved that the nanocomposite was amorphous. The high‐resolution TEM (HRTEM) image of a‐MoWS_x_/N‐RGO in Figure 1b does not show a lattice or Moiré pattern in the obtained materials, which demonstrated its amorphous nature. In addition, except for a broad peak at approximately 24°, which is typical for RGO,^[^ ^31^ ^]^ Figure 1c does not show a diffraction peak in the X‐ray diffraction (XRD) pattern, which further confirmed the amorphous structure of the nanocomposite. Medium‐resolution TEM and HRTEM images of a‐MoWS_x_/RGO@1:2–10 min and a‐MoWS_x_/RGO@2:1–10 min are shown in Figure S1a–d, Supporting Information. We observed that the structures of a‐MoWS_x_/RGO@1:2–10 min and a‐MoWS_x_/RGO@2:1–10 min were similar to that of a‐MoWS_x_/RGO@1:1–10 min. This result indicated that the catalysts prepared by plasma treatment in this work were lamellar and amorphous regardless of a change in the precursor ratio. We performed spectroscopic characterizations to investigate the elemental distribution. The energy dispersive X‐ray spectrum of a‐MoWS_x_/RGO@1:1–10 min is shown in Figure S2, Supporting Information. Figure 1d exhibits the high‐angle annular dark‐field scanning TEM (HAADF‐STEM) image and corresponding elemental mapping for a‐MoWS_x_/N‐RGO. The elemental mapping analysis proved that molybdenum, tungsten, and sulfur were homogeneously distributed on the RGO sheet and that nitrogen was successfully doped into RGO, which illustrated the feasibility and convenience of fabricating amorphous TMS/N‐RGO nanocomposites through simultaneous precursor reduction and GO doping by plasma technology.

### 2.2 HER Performance of a‐MoWS*_x_*/N‐RGO

**Figure 2:**
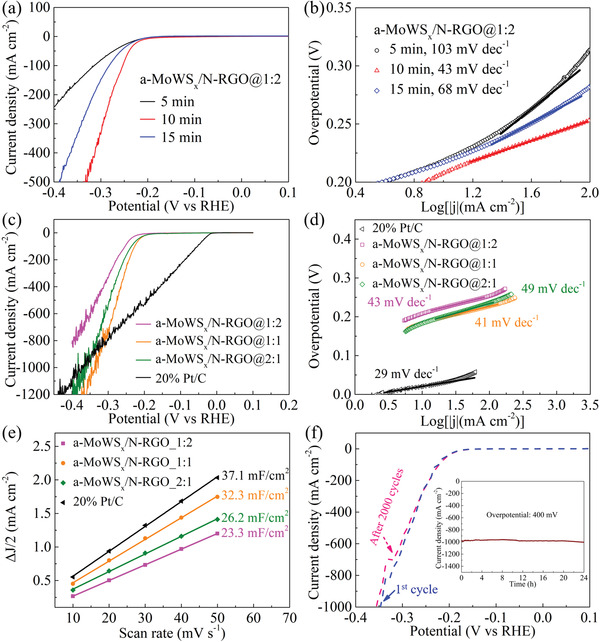
a) LSV polarization curves for a‐MoWS_x_/N‐RGO@1:2 samples prepared with various plasma treatment duration. b) Tafel slopes of the corresponding samples shown in (a). c) LSV polarization curves for a‐MoWS_x_/N‐RGO@1:2, a‐MoWS_x_/N‐RGO@1:1, and a‐MoWS_x_/N‐RGO@2:1 prepared with 10 min of plasma treatment and commercial 20 wt.% Pt/C. d) Tafel slopes for the corresponding samples shown in (c). e) The half capacitive current as a function of scan rate from 10 mV s^−1^ to 50 mV s^−1^. f) LSV curves measured for a‐MoWS_x_/N‐RGO@1:1–10 min before and after 2000 CV cycles at a scan rate of 50 mV s^−1^ in the potential range of 0.1 V to (−0.4) V (versus RHE) in 0.5 M H_2_SO_4_ (inset: CA curve at a constant overpotential of 400 mV).

We measured the linear sweep voltammetry (LSV) curves for a‐MoWS_x_/N‐RGO with various plasma treatment duration in 0.5 M H_2_SO_4_, and the results are shown in **Figure** **2**a and Figure S3, Supporting Information. Taking a‐MoWS_x_/N‐RGO@1:2 as an example, the overpotential at a current density of 100 mA cm^−2^ (*η*_100_) first decreased from 314 mV to 254 mV as the plasma treatment was increased from 5 min to 10 min and then bounced back to 283 mV when the plasma treatment duration reached 15 min. The Tafel slope for a‐MoWS_x_/N‐RGO@1:2 shown in Figure 2b follows a similar trend with *η*_100_ values of 103, 43, and 68 mV dec^−1^ for the 5, 10, and 15 min samples, respectively. The Tafel slopes of the three samples in Figure 2b suggest that the Volmer–Heyrovsky mechanism was operative.^[^ ^13^ ^]^ The rate‐determining steps of the HER catalyzed by the three samples, however, were different. The Tafel slope of the 5 min sample (103 mV dec^−1^) indicated that the Volmer reaction was the rate‐determining step, which implied slow HER kinetics. Conversely, the Tafel slopes of the 10 and 15 min samples (43 and 68 mV dec^−1^) suggested faster HER kinetics with the Heyrovsky reaction being the rate‐determining step.^[^ ^32^ ^]^ To explain why the rate‐determining step of the HER catalyzed by the 5 min sample was different from those catalyzed by 10 and 15 min samples, the electrochemical impedance spectra of a‐MoWS_x_/N‐RGO@1:2 with varied plasma treatment duration were recorded, and the results are shown in Figure S4, Supporting Information. It can be seen from Figure S4, Supporting Information, that the charge transfer resistance (*R*_ct_) of the 5 min sample was larger than those of 10 and 15 min samples. This indicated that, in our experiment, a plasma duration of 5 min was too short to obtain RGO with good conductivity. The low conductivity of RGO in the 5 min sample limited charge transport from the glassy carbon electrode to the amorphous molybdenum tungsten sulfide, which resulted in the discharge step (Volmer reaction) being the rate‐determining step. On the other hand, the conductivity of RGO in 10 and 15 min samples was improved because of the reducing and doping effect of Ar + NH_3_ plasma. So, the rate‐determining steps of the HER catalyzed by these two samples were no longer the Volmer reaction but the electrochemical desorption step (Heyrovsky reaction). These results indicated that plasma treatment could accelerate the HER kinetics of the a‐MoWS_x_/N‐RGO catalysts. The HER activity of a‐MoWS_x_/N‐RGO@1:1 and a‐MoWS_x_/N‐RGO@2:1 also exhibited the same trend with increasing plasma treatment duration. These results suggested that the reaction kinetics for the plasma treatment proposed for unary amorphous molybdenum sulfide in our prior work^[^ ^33^ ^]^ can be extended to the alloyed amorphous molybdenum tungsten sulfide system. Because Ar + NH_3_ plasma has both a strong reducing and etching effect,^[^ ^34^ ^]^ to balance the conversion and etching effect and obtain presumably maximal active sulfur species in a‐MoWS_x_/N‐RGO, we experimentally proved 10 min of plasma treatment to be the optimal duration. Furthermore, we also recorded the LSV polarization curves of a‐MoS_x_/N‐RGO and a‐WS_x_/N‐RGO in 0.5 M H_2_SO_4_, and the results are shown in Figure S5, Supporting Information. Figure S5, Supporting Information, shows that the HER activity of the alloyed a‐MoWS_x_/N‐RGO under large current densities was notably improved compared with those of a‐MoS_x_/N‐RGO and a‐WS_x_/N‐RGO. This result further demonstrated the effectiveness of our tungsten‐doping strategy to boost the HER activity of a‐MoS_x_ under high currents.

Except for changing the duration of the plasma treatment, the HER activity of a‐MoWS_x_/N‐RGO could be tuned by adjusting the precursor ratio of (NH_4_)_2_MoS_4_ to (NH_4_)_2_WS_4_. As shown in Figure 2c,d, when the plasma treatment duration was fixed to 10 min, the HER catalytic performance of a‐MoWS_x_/N‐RGO could be further improved by increasing the mass ratio of (NH_4_)_2_MoS_4_ to (NH_4_)_2_WS_4_ to 1:1 (i.e., a‐MoWS_x_/N‐RGO@1:1). When the precursor ratio reached 2:1, however, the electrocatalytic performance of a‐MoWS_x_/N‐RGO@2:1 began to decline. Notably, at 850 mA cm^−2^, the overpotential for a‐MoWS*_x_*/N‐RGO@1:1 became lower than that of the 20 wt.% Pt/C catalyst (Tanaka, Tokyo, Japan). More interesting, at a large current density of 1000 mA cm^−2^, the a‐MoWS*_x_*/N‐RGO@1:1–10 min catalyst exhibited an overpotential of 348 mV, which was lower than that of 391 mV obtained over the commercial 20 wt.% Pt/C. To the best of our knowledge, such HER activity for this catalyst at high current densities outperformed that of all previously reported heteroatom‐doped MoS_2_‐based catalysts, and it is the second‐highest one among all MoS_2_‐based catalysts (see Table S1, Supporting Information). Figure S6a, Supporting Information, which provides an enlarged view of the low overpotential region in Figure 2c, shows that the trend of the HER activity at low current densities was similar to that at high current densities. Unlike the HER on the 20 wt.% Pt/C catalyst, which started immediately when the cathodic potential was applied but proceeded relatively slowly in the high current region, the HER on our a‐MoWS_x_/N‐RGO started slowly initially, but as the overpotential increased, it proceeded rapidly with very high current densities. To explore the reason for this high HER activity of a‐MoWS_x_/N‐RGO@1:1–10 min, we estimated the electrochemical active surface area (ECSA) from the double‐layer capacitance (*C*_dl_) shown in Figure 2e. We calculated the *C*_dl_ values for a‐MoWS_x_/N‐RGO@1:2, a‐MoWS_x_/N‐RGO@1:1, a‐MoWS_x_/N‐RGO@2:1, and commercial Pt/C to be 23.3, 32.3, 26.2, and 37.1 mF cm^−2^, respectively. This indicated that an appropriate ratio of (NH_4_)_2_MoS_4_ to (NH_4_)_2_WS_4_ in the precursor yielded the highest electrochemical active area among all samples and the obtained value was comparable to that of 20 wt.% Pt/C. Interestingly, although the ECSA of a‐MoWS_x_/N‐RGO@1:1 (78.7 ${\mathrm{cm}}_{\mathrm{ECSA}}^{2}$) was lower than that of Pt/C (90.4 ${\mathrm{cm}}_{\mathrm{ECSA}}^{2}$), the ECSA‐normalized LSV curves (Figure S7, Supporting Information) suggested that the intrinsic (instead of surface‐area‐related) activity of a‐MoWS_x_/N‐RGO@1:1 beyond an overpotential of 295 mV was appreciably higher than that of Pt/C. Similar methods for estimating the intrinsic activity of a catalyst have also been reported in previous studies.^[^ ^35^, ^36^ ^]^ This result corroborated the conclusion of a‐MoWS_x_ being more active toward HER than platinum at high current densities from density functional theory (DFT) calculations as described later in this paper. We also performed electrochemical impedance spectroscopy (EIS) to investigate the electrode kinetics during the HER. The Nyquist plots for these electrodes are shown in Figure S6b, Supporting Information. We obtained the *R*_ct_ related to the interfacial charge transfer process from the diameters of the semicircles in the high‐frequency zone. To precisely compare the *R*_ct_ of these samples, we fitted the EIS results using the equivalent circuit shown in Figure S8, Supporting Information.^[^ ^37^ ^]^ As shown in Figure S6b, Supporting Information, the *R*_ct_ of the a‐MoWS_x_/N‐RGO@1:1 sample was smaller (14.1 Ω) than that of the a‐MoWS_x_/N‐RGO@1:2 (26.2 Ω) and a‐MoWS_x_/N‐RGO@2:1 (18.8 Ω) samples, which demonstrated its efficient interfacial electron‐transfer kinetics over a‐MoWS_x_/N‐RGO@1:1. In addition, the precursor ratio had an impact on the microscopic morphology. The SEM images of a‐MoWS_x_/N‐RGO@1:2, a‐MoWS_x_/N‐RGO@1:1, and a‐MoWS_x_/N‐RGO@2:1 are shown in Figure S9a–c, Supporting Information. As shown in Figure S9a–c, Supporting Information, the humps and wrinkles in a‐MoWS_x_/N‐RGO@1:1 were denser than those in a‐MoWS_x_/N‐RGO@1:2 and a‐MoWS_x_/N‐RGO@2:1, which contributed to the higher *C*_dl_ value obtained for a‐MoWS_x_/N‐RGO@1:1 than that of a‐MoWS_x_/N‐RGO@1:2 and a‐MoWS_x_/N‐RGO@2:1.

Long‐term stability is another critical criterion necessary to evaluate the electrocatalytic performance of HER catalysts for large‐scale applications in water electrolysis. We performed an accelerated degradation test (ADT) and chronoamperometry (CA) to evaluate the catalytic stability. For the ADT, we conducted 2000 cycles of cyclic voltammetric scans between 0 and −0.4 V (versus RHE) and compared the final and initial polarization curves. As shown in Figure 2f, the overpotentials at high current densities showed a mere 2% negative shift (e.g., the initial overpotential at 1000 mA cm^−2^ was 349 mV, and the final value was 356 mV, which represented an increase of 2%). This shift may have been due to the slight peeling‐off of the catalyst from the electrode surface during rapid bubble emission, and the parts below 350 mA cm^−2^ in the first and final polarization curves almost overlapped. Additionally, the a‐MoWS_x_/N‐RGO@1:1–10 min catalyst could be operated steadily at large current densities of around 1000 mA cm^−2^: this activity could be well maintained after continuous 24 h testing (Figure 2f, inset), which makes it a potential candidate for practical application in industrial hydrogen production. The possibility of platinum deposition on the a‐MoWS_x_/N‐RGO@1:1–10 min catalyst was eliminated by inductively coupled plasma optical emission spectroscopy (ICP‐OES). The platinum content in the sample after the durability test was below the detection limit. The XRD pattern (Figure S10a, Supporting Information), TEM images (Figure S10b,c, Supporting Information), and HAADF‐STEM image and elemental mapping (Figure S10d, Supporting Information) for a‐MoWS_x_/N‐RGO@1:1–10 min showed no change in the amorphous structure, lamellar morphology, or composition after the durability test, which provided further evidence of its good stability. We measured the element content of a‐MoWS*_x_*/N‐RGO@1:1–10 min before and after the stability test by ICP‐OES, and the results are shown in Table S2, Supporting Information. The element content was well maintained after the stability test, thus eliminating the possibility of element dissolution. Utilizing a‐MoWS_x_/N‐RGO@1:1–10 min as the cathode and RuO_2_ as the anode, an acidic whole cell water electrolyzer was assembled (details in the Supporting Information). The polarization curve of the a‐MoWS_x_/N‐RGO@1:1–10 min–RuO_2_ couple was recorded and compared to the cell constructed using 20 wt.% Pt/C (cathode) and RuO_2_ (anode), both without iR‐compensation. As shown in Figure S11, Supporting Information, the a‐MoWS_x_/N‐RGO@1:1–10 min–RuO_2_ couple exhibited superior water splitting performance than the Pt/C‐RuO_2_ couple at high current densities, demonstrating the feasibility of utilizing the a‐MoWS_x_/N‐RGO as an efficient industrial HER catalyst. These electrochemical results proved that a‐MoWS_x_/N‐RGO@1:1–10 min exhibited decent HER performance at industrial‐level current densities, including small overpotentials and small Tafel slopes, and exhibited good durability in acidic media.

### 2.3 Mechanism Discussion for a‐MoWS_x_/N‐RGO

To reveal the mechanism for enhancing the HER performance of a‐MoWS_x_ through compositional tuning, we analyzed the surface chemical states and chemical composition of the samples by XPS and ICP‐OES. The high‐resolution molybdenum 3d XPS spectra for a‐MoWS_x_/N‐RGO@1:2, a‐MoWS_x_/N‐RGO@1:1, and a‐MoWS_x_/N‐RGO@2:1 with a fixed duration of plasma treatment of 10 min are shown in Figure S12, Supporting Information. The molybdenum 3d spectra were fitted to four contributions: the doublet observed at binding energies of 228.2 and 231.4 eV corresponded to Mo^4+^ 3d_5/2_/3d_3/2_; the doublet located at 230.6 and 233.7 eV corresponded to Mo^5+^ 3d_5/2_/3d_3/2_; the doublet shown at 233.1 and 236.2 eV corresponded to Mo^6+^ 3d_5/2_/3d_3/2_; and the peak at approximately 227.2 eV was attributed to sulfur 2s electrons.^[^ ^38^, ^39^, ^40^ ^]^ The tungsten 4f spectra shown in Figure S13, Supporting Information, were deconvoluted into three doublets: the doublet located at 32.9 and 35.0 eV corresponded to W^4+^ 4f_7/2_/4f_5/2_; the doublet located at 33.3 and 35.3 eV corresponded to W^5+^ 4f_7/2_/4f_5/2_; and the doublet located at 36.1 and 38.5 eV corresponded to W^6+^ 4f_7/2_/4f_5/2_.^[^ ^39^, ^41^ ^]^ The molybdenum 3d and tungsten 4f spectra indicated that the molybdenum and tungsten atoms in a‐MoWS_x_/N‐RGO existed in a mixed oxidation state, which is typical for a‐TMS.^[^ ^22^, ^41^, ^42^, ^43^ ^]^ As shown in Figure S14, Supporting Information, the core‐level sulfur 2p spectra are composed of two doublets: the two peaks at 162.2 and 163.3 eV were assigned to the sulfur 2p_3/2_ and sulfur 2p_1/2_ electrons of S^2 −^ , respectively, and the two peaks at 163.8 and 164.9 eV were attributed to the sulfur 2p_3/2_ and sulfur 2p_1/2_ electrons of $S_{2}^{2-}$, respectively.^[^ ^22^, ^38^, ^40^ ^]^ In our prior work, we had demonstrated that $S_{2}^{2-}$ species in amorphous MoS_x_ possessed higher catalytic activity toward the HER than S^2 −^ species.^[^ ^33^ ^]^ Whether or not this conclusion pertains to the alloyed state of amorphous MoWS_x_ remains unexamined. In addition, although the content of $S_{2}^{2-}$ species in a‐MoWS_x_/N‐RGO@1:1 was higher than that in a‐MoWS_x_/N‐RGO@1:2 and a‐MoWS_x_/N‐RGO@2:1 (Table S3, Supporting Information), this did not explain why the a‐MoWS_x_/N‐RGO@1:1 sample exhibited excellent HER activity at high current densities.

**Figure 3:**
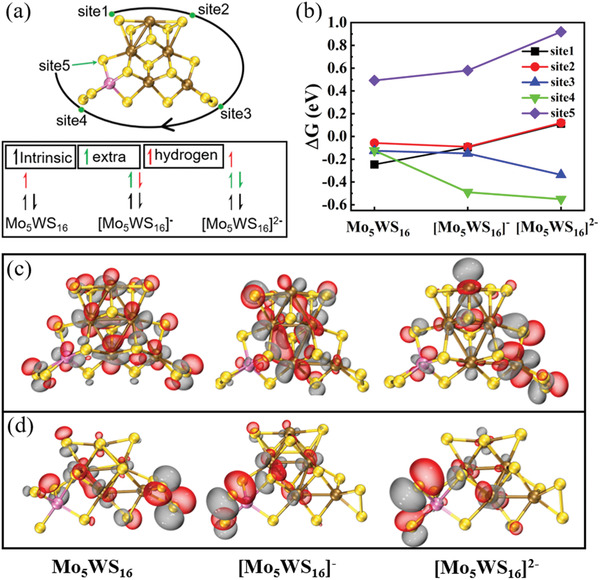
Analysis of the hydrogen evolution performance of Mo_5_WS_16_ clusters with possible sites and varying number of extra charges with a) H‐adsorption sites and electronic configurations under different circumstances and b) $\Delta G_{H^{*}}$ in neutral and electron‐injected clusters. Transition‐state orbital isosurface occupied by hydrogen before adsorption in c) 2S^2 −^ and d) $S_{2}^{2-}$. All isosurface values are identical.

To further clarify why the a‐MoWS_x_/N‐RGO@1:1 catalyst exhibited superior HER activity, especially at large current densities, we systematically performed DFT calculations. Considering the atomic percentages of molybdenum, tungsten, and sulfur and the relative percentages of S^2 −^ and $S_{2}^{2-}$ (Tables S3 and S4, Supporting Information), we selected three cluster models—Mo_5_WS_16_, Mo_3_W_3_S_16_, and MoW_5_S_16_ (Figure S15, Supporting Information)—as representatives of a‐MoWS_x_/N‐RGO@1:2, a‐MoWS_x_/N‐RGO@1:1, and a‐MoWS_x_/N‐RGO@2:1, respectively. As shown in Table S4, Supporting Information, the atomic ratios in the DFT models were not strictly the same as those from the experiments; however, it did not affect the trend of the HER performance of the Mo—W—S clusters and the underlying mechanisms, as described next. All three models contained the three types of potentially active sites: bridging S^2 −^ (one single S^2 −^ atom connecting to transition metal atoms), double S^2 −^ (2S^2 −^ , two S^2 −^ atoms connecting to two identical transition metal atoms), and terminal $S_{2}^{2-}$ (a pair of sulfur atoms bonded to each other and connected to one transition metal atom), which were classified by S—S interaction with an electronic localization function (Figure S15, Supporting Information). Previous studies have shown that the HER activity of $S_{2}^{2-}$ has been better than that of bridging S^2 −^ .^[^ ^33^, ^44^ ^]^ Nevertheless, we could not find any reports concerning the HER activity of 2S^2 −^ in the literature. For the Mo_5_WS_16_ cluster, 2S^2 −^ was located at site 1 and site 2, whereas $S_{2}^{2-}$ was located at site 3 and site 4, and site 5 contained a S^2 −^ (**Figure** **3**a). Spin multiplicity test results showed that neutral clusters did not contain any single electron before adsorbing the hydrogen atoms (Figure 3a), which guaranteed that the electronic configuration of the cluster was in the ground state rather than the excited state. The Gibbs free energies of hydrogen adsorption ($\Delta G_{H^{*}}$) for hydrogen adsorbed to the three types of potentially active sites are shown in Figure 3b. For site 1 and site 2 (both sites belong to 2S^2 −^ ) in neutral Mo_5_WS_16_, the $\Delta G_{H^{*}}$ was not identical. Tungsten doping shortened the S—S distance of 2S^2 −^ in site 2 (site 1 versus site 2, 2.79 versus 2.58 Å in Figure S15, Supporting Information). When the hydrogen atom was absorbed at site 1, 2S^2 −^ in site 2 transformed to $S_{2}^{2-}$ because the S—S distance was close to 2.35 Å. When the hydrogen atom was absorbed at site 2, the S—S distance of 2S^2 −^ at site 1 was distant enough that 2S^2 −^ in site 1 would not transform to $S_{2}^{2-}$. The absence of a redundant bonding process in site 1 during the hydrogen‐adsorbed process at site 2 produced a larger electronic energy (internal energy) in the cluster. Thus, a $\Delta G_{H^{*}}$ at site 2 closer to zero than site 1 was obtained. Note that site 2 was a typical 2S^2 −^ catalytic active site. At sites 3 and 4, $S_{2}^{2-}$ presented a chemical bond‐breaking process after adsorbing hydrogen. The cleavage of the S—S bond in $S_{2}^{2-}$ introduced an extra 0.031 eV of entropy and 0.035 eV of electronic energy (internal energy) after hydrogen adsorption, showing a more negative $\Delta G_{H^{*}}$—that is, approximately 0.066 eV more than that of 2S^2 −^ (site 2) in the Mo_5_WS_16_ neutral cluster. In fact, the smaller the change in entropy and activation energy after adsorption, the more conducive the conditions were to the hydrogen‐evolution reaction. In bridging S^2 −^ at site 5, the two unpaired electrons outside the sulfur atom were strongly bonded with molybdenum and tungsten, so that there was no suitable vacancy to accept the hydrogen atom, compared with 2S^2 −^ . In the Mayer bond order analysis, the former (1.663 + 0.638 = 2.301) was much stronger than the later (0.842 + 1.091 = 1.933), which resulted in a more difficult hydrogen‐bonding process. Thus, site 5 exhibited inferior HER activity in a neutral state. Overall, the best active catalytic species under a small current density were 2S^2 −^ and $S_{2}^{2-}$.

Another important issue to address was the change in $\Delta G_{H^{*}}$ at different sites after charge injection, which mimicked the situation of high current densities.^[^ ^45^ ^]^ In this case, because of the aggregated negative charges on the electrode and the increased electrode potential, some electrons could likely overcome the island effect in quantum confinement and drill into clusters. Clusters with one extra electron could be qualitatively considered as the dividing crest between a high and a low current density because the two charges had to overcome a potential barrier several times greater than that for one charge. The electronic configuration of charged clusters could be determined according to the neutral clusters and the Pauli exclusion principle (Figure 3a). Extra electrons occupied the lowest unoccupied frontier orbital, which led to a small or large shift in the electron cloud. This shift could change the ability of the catalyst to adsorb (or desorb) hydrogen. The change in $\Delta G_{H^{*}}$ in Mo_5_WS_16_ with various injected charges is shown in Figure 3b. Bridging S^2 −^ had a sharp increase in terms of $\Delta G_{H^{*}}$, and its performance deteriorated at a high current density in [Mo_5_WS_16_]^2−^ (Mo_5_WS_16_ + 2e^−^). For 2S^2 −^ , site 1 and site 2 showed the same catalytic performance after the S—S distance in site 2 increased because of electron injection. Taking 2S^2 −^ (site 2) as an example, more extra electrons were distributed on 2S^2 −^ in [Mo_5_WS_16_]^−^, corresponding to an easier way to adsorb hydrogen than in Mo_5_WS_16_, although this was more difficult in [Mo_5_WS_16_]^2−^ because extra electrons effectively occupied the lowest unoccupied frontier orbital of 2S^2 −^ , as shown in the transition‐state molecular orbital isosurface graph (Figure 3c). For transition state $S_{2}^{2-}$ (e.g., site 4), the situation was reversed: injected electrons led to an increased localization of the molecular orbital on $S_{2}^{2-}$ (Figure 3d), which resulted in an easier hydrogen adsorption (i.e., more difficult to desorb) with an increasingly negative $\Delta G_{H^{*}}$. In molecular orbital theory, the bonding process of hydrogen absorption could be regarded as the transfer process of isolated localized electrons on hydrogen atoms to delocalized electrons in the empty orbital of the Mo—W—S clusters. This enthalpy change could be affected by electron pairing and orbital energy. Notably, this bonding process was the transition state of a site rather than the ground state of the cluster, and the transition states were different for different sites. Thus, Figure 3c,d was applicable only to sites 2 and 4, respectively. Among the three types of potentially active sites (S^2 −^ , $S_{2}^{2-}$, and 2S^2 −^ ), only 2S^2 −^ exhibited a negative to positive fluctuation in $\Delta G_{H^{*}}$ around 0 eV with electron injection, because the electrons preferentially filled around 2S^2 −^ (Figure 3b). These results showed that 2S^2 −^ possessed an ability to withstand large current density and better hydrogen‐evolution performance.

**Figure 4:**
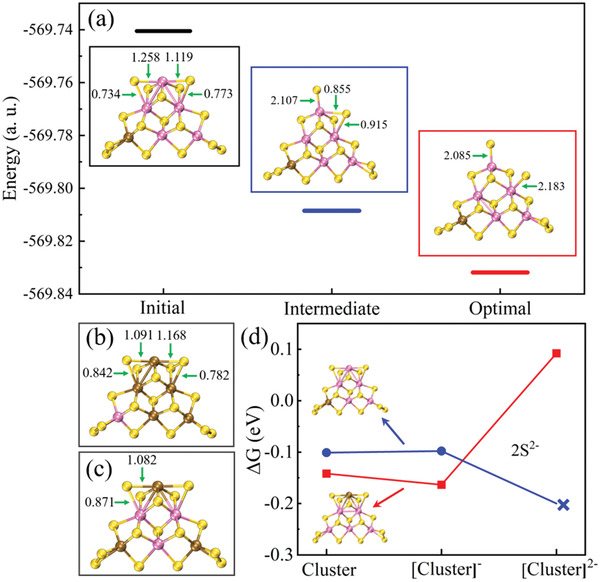
Stability analysis and hydrogen evolution performance of Mo—W clusters, with a) dynamic structural changes caused by injection of two additional electrons marked with the Mayer bond order in [MoW_5_S_16_]^2−^ and Mayer bond order in stable b) [Mo_5_WS_16_]^2−^ and c) [Mo_3_W_3_S_16_]^2−^ clusters. d) Hydrogen evolution performance of stable (red) and unstable (blue) [MoW_5_S_16_]^2−^ with a varying number of extra electrons.

On the other hand, charge injection also affected the stability of cluster structures. Under a high current density, the structure in the [MoW_5_S_16_]^2−^ cluster could be distorted. Immediately after injection of the second electron, the strength of the metal‐2S^2 −^ bond shifted laterally by approximately 0.524 (in the Mayer bond order; see **Figure** **4**a). Soon, this large deviation led to the fracture and reorganization of 2S^2 −^ , splitting into a bridging S^2 −^ and a terminal S^2 −^ with an extra electron because the Mayer bond order of the latter reached a value of approximately 2.107 (Figure 4a). Although one electron reached the exact location, the Mayer bond order on the other side also produced the same deviation. Thus, the active 2S^2 −^ groups were broken in [MoW_5_S_16_]^2−^ (Figure 4a). This resulted in the exhibition of worse catalytic activity compared with [Mo_5_WS_16_]^2−^, which was consistent with the experimental results. This structural damage was related to the tungsten (or molybdenum) sites. Because the distance between sulfur and the top metal element (2S^2 −^ ‐T) was shorter than that of the edge‐metal element (2S^2 −^ ‐E) in 2S^2 −^ (Figure S15, Supporting Information) and the electronegativity of tungsten was greater than that of molybdenum (2.36 versus 2.16), the difference became weaker or stronger when 2S^2 −^ bonded with tungsten and molybdenum, respectively. If the top metal element was molybdenum (2S^2 −^ ‐T(Mo)), the interaction of 2S^2 −^ ‐T was weaker and 2S^2 −^ ‐E became stronger, balancing the intrinsically different distances between the two and resulting in a better stable structure. Instead, 2S^2 −^ ‐T(W) exacerbated the shift in the electrons located far away from 2S^2 −^ ‐E(Mo) and led to the breakup of 2S^2 −^ ‐E(Mo). The Mayer bond orders for [Mo_5_WS_16_]^2−^ (Figure 4b) and [Mo_3_W_3_S_16_]^2−^ (Figure 4b,c) between 2S^2 −^ ‐T and 2S^2 −^ ‐E were closer than those shown in Figure 4a, which induced stable structures. The stable structure under high current density accounted for one‐third of the total structures in the MoW_5_S_16_ system (Figure S16, Supporting Information), whose catalytic performance was slightly superior to [Mo_5_WS_16_]^2−^ ([MoW_5_S_16_]^2−^ versus [Mo_5_WS_16_]^2−^, 0.09 eV versus 0.12 eV; see Figure 4d) because the energy in [MoW_5_S_16_]^2−^ was more stable (~0.026 eV lower than [Mo_5_WS_16_]^2−^; see Figure S17, Supporting Information).

**Figure 5:**
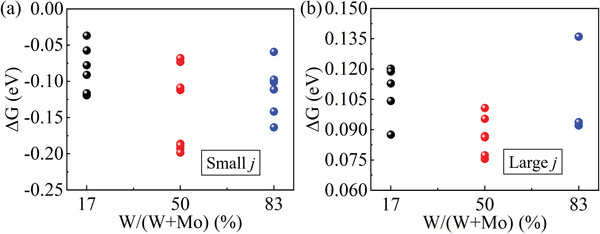
Gibbs free energies for all possible Mo—W structures under a) a small current density and b) a large current density with varying tungsten content.

Thus, Mo—W clusters containing a low tungsten content possessed better catalytic performance under a small current density, whereas extremely high‐content tungsten clusters could be used to obtain better hydrogen evolution activity under a high current density despite serious structural damage with extra electrons. If the tungsten content could be controlled to separate this undesirable situation and make 2S^2 −^ ‐T(W) appears with a small probability, then better catalytic activity under a high current density could be expected. For this purpose, we employed the Mo_3_W_3_S_16_ clusters to verify this idea. All possible tungsten‐occupied‐site structures in different tungsten‐content clusters were calculated. Under a small current density, Mo_3_W_3_S_16_ and MoW_5_S_16_ showed worse catalytic activity than Mo_5_WS_16_ because of a more negative $\Delta G_{H^{*}}$, which resulted from the lower electronic structure energy obtained after adsorbing hydrogen atoms (**Figure** **5**a), which was also observed in the LSV curves for a‐MoWS_x_/N‐RGO in the low overpotential region (Figure S6a, Supporting Information). This situation was completely reversed under high current density. According to the stability characteristics of the Mo—W structure discussed earlier for 2S^2 −^ ‐T and 2S^2 −^ ‐E, more than half of all possible [Mo_3_W_3_S_16_]^2−^ structures could maintain good stability (Figure S18, Supporting Information). In addition, the structure with lower energy resulted in better catalytic activity for these clusters under a high current density (Figure 5b).

Finally, the $\Delta G_{H^{*}}$ of platinum with and without charge injection are shown in Table S5, Supporting Information, for comparison. It can be seen that platinum was more active toward the HER than a‐MoWS_x_ in a neutral state, but this situation would be reversed after charge injection. This result was in good agreement with the trend of intrinsic activity shown in the ECSA‐normalized LSV curves (Figure S7, Supporting Information).

## 3 Conclusion

We developed a facile route for synthesizing a‐MoWS_x_/N‐RGO nanocomposites as highly efficient catalysts for the HER at high current density using plasma treatment. The performance of a‐MoWS_x_/N‐RGO was optimized by adjusting the plasma treatment duration and chemical composition. The optimal a‐MoWS_x_/N‐RGO@1:1–10 min sample reached a high current density of 1000 mA cm^−2^ at a low overpotential of 348 mV, which outperformed not only a commercial 20 wt.% Pt/C but also all heteroatom‐doped MoS_2_‐based catalysts reported to date. The a‐MoWS_x_/N‐RGO@1:1–10 min sample also exhibited good long‐term stability at an industrial‐level current density of ~1000 mA cm^−2^. The 2S^2 −^ species was able to maintain high activity with electron injection because extra electrons preferentially filled at 2S^2 −^ , which contributed greatly to exceptional HER performance under high current densities. The introduction of tungsten tended to lower the electronic structure energy, resulting in a closer‐to‐zero positive $\Delta G_{H^{*}}$. Excessive tungsten introduction, however, could lead to structural damage and trigger worse HER performance under high current densities. This work shed light on designing and fabricating highly efficient HER catalysts that can be used for practical applications of industrial hydrogen production.

## Conflict of Interest

The authors declare no conflict of interest.

## Supporting information

Supporting Information

## Data Availability

The data that support the findings of this study are available from the corresponding author upon reasonable request.
