# Supplementary material for: Boosting Hydrogen Evolution Reaction Activity of Amorphous Molybdenum Sulfide Under High Currents Via Preferential Electron Filling Induced by Tungsten Doping
Source: Adv Sci (Weinh). 2022 Jul 25;9(27):2202445. doi: 10.1002/advs.202202445 (PMC9507386; doi:10.1002/advs.202202445)
Supplement: Supplementary file 1 — Supporting Information [file ADVS-9-2202445-s001.pdf]

ADVANCED SCIENCE
Open Access

Supporting Information

for *Adv. Sci.*, DOI 10.1002/advs.202202445

Boosting Hydrogen Evolution Reaction Activity of Amorphous Molybdenum Sulfide Under High Currents Via Preferential Electron Filling Induced by Tungsten Doping

Dai Zhang, Feilong Wang, Wenqi Zhao, Minghui Cui, Xueliang Fan, Rongqing Liang, Qiongrong Ou and Shuyu Zhang**

Supporting Information

Boosting Hydrogen Evolution Reaction Activity of Amorphous Molybdenum Sulfide under High Currents via Preferential Electron Filling Induced by Tungsten Doping

Dai Zhang, Feilong Wang, Xueliang Fan, Wenqi Zhao, Minghui Cui, Rongqing Liang, Qiongrong Ou* and Shuyu Zhang*

D. Zhang, R. Liang, Q. Ou, S. Zhang

Institute of Future Lighting, Academy for Engineering and Technology, Fudan University, Shanghai 200433, China PR

Email: qrou@fudan.edu.cn (Q. O.) and zhangshuyu@fudan.edu.cn (S. Z.)

F. Wang, W. Zhao, M. Cui, R. Liang, Q. Ou, S. Zhang

Institute for Electric Light Sources, School of Information Science and Technology, Fudan University, Shanghai 200433, China PR

X. Fan

Department of Chemistry, Shanghai Key Laboratory of Molecular Catalysis and Innovative Materials, Laboratory of Advanced Materials and Collaborative Innovation Center of Chemistry for Energy Materials, Fudan University, Shanghai 200433, China PR

D. Zhang and F. Wang contributed equally to this work.

Content

Experimental Section 4

Figure S1 The medium and high-resolution TEM images of (a, b) a-$MoWS_x$/RGO@1:2-10 min and (c, d) a-$MoWS_x$/RGO@2:1-10 min. 8

Figure S2 The EDX spectrum of a-$MoWS_x$/RGO@1:1-10 min. 9

Figure S3 (a) LSV polarization curves for a-$MoWS_x$/N-RGO@1:1 with varied plasma treatment time. (b) LSV polarization curves for a-$MoWS_x$/N-RGO@2:1 with varied plasma treatment time. 10

Figure S4 Nyquist plots of the electrochemical impedance spectra of a-$MoWS_x$/N-RGO@1:2 with varied plasma treatment duration. 11

Figure S5 LSV polarization curves of a-MoS_x/N-RGO, a-WS_x/N-RGO and a-$MoWS_x$/N-RGO in 0.5 M H_2SO_4. 12

Figure S6 (a) Enlarged view of low overpotential region in Fig 2c. (b) Nyquist plots of the electrochemical impedance spectra. 13

Figure S7 Polarization curves from Figure 2c normalized to the electrochemical surface area (ECSA)...14

Figure S8 The equivalent circuit model for EIS response fitting. 15

Figure S9 SEM images of (a) a-$MoWS_x$/N-RGO@1:2, (b) a-$MoWS_x$/N-RGO@1:1, (c) a-$MoWS_x$/N-RGO@2:1. 16

Figure S10 Structural, morphological and elemental characterization of the catalyst after the long-term durability test. 17

Figure S11 LSV comparing the water-splitting performance of a-$MoWS_x$/N-RGO@1:1-RuO_2 couple and Pt/C-RuO_2 couple 18

Figure S12 High-resolution Mo 3d XPS spectra of a-$MoWS_x$/N-RGO with varied precursor ratio and a fixed plasma treatment time of 10 min. 19

Figure S13 High-resolution W 4f XPS spectra of a-$MoWS_x$/N-RGO with varied precursor ratio and a fixed plasma treatment time of 10 min. 20

Figure S14 High-resolution S 2p XPS spectra of a-$MoWS_x$/N-RGO with varied precursor ratio and a fixed plasma treatment time of 10 min. 21

Figure S15 Three specific cluster models with different W-content at a random site....................22

Figure S16 All possible structures of $[MoW_5S_{16}]^{2-}$ with unstable geometry (a) and (b), and stable geometry (c) and (d)...23

Figure S17 Electronic energy difference between $[Mo_5WS_{16}]^{2-}$ (black) and $[MoW_5S_{16}]^{2-}$ (red). ...24

Figure S18 All possible structures of $[Mo_3W_3S_{16}]^{2-}$ with stable geometry (a) - (g), and unstable geometry (h) - (l)..25

Table S1. Comparison of the performances of MoS_2-based catalysts toward high-current-density HER in 0.5 M H_2SO_4. 26

Table S2. ICP-OES results for a-$MoWS_x$/N-RGO@1:1-10 min before and after the stability test.27

Table S3. The deconvolution results of core-level S 2p spectra of the final samples with varied precursor ratio. 27

Table S4. The atomic content of Mo, W and S in the final samples with varied precursor ratio. ...27

Table S5. Comparison of Gibbs free energies of a-$MoWS_x$ and Pt crystal. 27

References...28

Experimental Section

Preparation of a-MoWS$_x$/N-RGO

In our previous work,[1] we have demonstrated that, in amorphous molybdenum sulfide/reduced graphene oxide (a-MoS$_x$/RGO) system, a reasonable amount of RGO is essential for improving the efficiency of charge transfer and the overall HER performance, and that the optimal mass precursor ratio of ammonium tetrathiomolybdate ($(NH_4)_2MoS_4$) to graphene oxide (GO) was 3:1. In this work, the mass ratio of total amount of ammonium tetrathiomolybdate (Sigma Aldrich, 99.97%, USA) and ammonium tetrathiotungstate ($(NH_4)_2WS_4$, Sigma Aldrich, 99.9%, USA) to GO was fixed to 3:1. Homogeneous GO/ N,N-Dimethylformamide (DMF) suspensions (1 mg mL^{-1}) were prepared by sonicating GO powder (10 mg, XFNANO Materials Tech Co., Ltd., China) in DMF (10 ml, Sinopharm Chemical Reagent Co., Ltd., China) for 30 min. N-RGO has two functions: 1) preventing a-MoWS$_x$ from agglomerating; 2) enhancing the conductivity of a-MoWS$_x$.[2, 3] To study the influence of mass ratio of $(NH_4)_2MoS_4$ to $(NH_4)_2WS_4$ on HER activity of the resultant catalysts, controlled experiments were conducted by adjusting the $(NH_4)_2MoS_4$ to $(NH_4)_2WS_4$ ratio: $(NH_4)_2MoS_4$ (10 mg) and $(NH_4)_2WS_4$ (20 mg), $(NH_4)_2MoS_4$ (15 mg) and $(NH_4)_2WS_4$ (15 mg), $(NH_4)_2MoS_4$ (20 mg) and $(NH_4)_2WS_4$ (10 mg), were added into three individual GO suspensions (1 mg mL^{-1}), respectively. For structural characterization, a droplet of suspension (40 μl) was drop-cast onto a glass substrate, and the solvent was allowed to dry overnight. For electrochemical measurements, a droplet of suspension (5 μl) containing 20 μg precursor (GO + $(NH_4)_2MoS_4$ + $(NH_4)_2WS_4$) was drop-cast onto a glassy carbon electrode (GCE) with a diameter of 3 mm, and the solvent was allowed to evaporate in air. The catalyst loading on the working electrode was ~0.283 mg cm^{-2}. Plasma treatment was carried out using a custom-made radio frequency inductively coupled plasma (ICP) system at room temperature, and its detailed configuration can be found in our previous work.[4] The plasma was induced by a copper coil, which was powered by a 13.56 MHz radio frequency source. A mechanical pump and a turbomolecular pump were used to generate a base vacuum of 1.0×10^{-3} Pa. An overall gas feed of 100 standard cubic centimeters per minute (sccm) containing 90 sccm for Ar and 10 sccm for NH_3 was introduced through mass flow controllers. The specimens were treated with plasma under 4 Pa at a power of 90 W, for a varied treatment duration of 5 min, 10 min and 15 min. The

as-fabricated samples were named a-$MoWS_x$/N-RGO@XX-YY (where XX denotes the ratio of $(NH_4)_2MoS_4$ to $(NH_4)_2WS_4$ and YY denotes the plasma treatment time). For comparison, a-MoS_x/N-RGO and a-WS_x/N-RGO were prepared via the identical method for fabricating a-$MoWS_x$/N-RGO except for using $(NH_4)_2MoS_4$ or $(NH_4)_2WS_4$ alone, respectively. The LSV polarization curves of a-MoS_x/N-RGO and a-WS_x/N-RGO in 0.5 M H_2SO_4 are shown in Figure S4.

Characterization of a-$MoWS_x$/N-RGO nanocomposites

The morphologies of the samples were characterized by transmission electron microscopy (TEM, FEI Talos F200X G2) and field-emission scanning electron microscopy (FESEM, Nova NanoSem 450). Elemental mapping via energy dispersive spectroscopy (EDS) was performed during TEM analysis. X-ray diffraction (XRD) patterns were recorded using a Bruker D8 Advance X-ray diffractometer with a Cu K_α X-ray radiation source. X-ray photoelectron spectroscopy (XPS) was conducted on a ESCALAB Xi+ spectrometer (ThermoFischer, USA). The XPS deconvolution processing was conducted on Thermo Avantage software and peak energies of all elements were calibrated by assigning the major C 1s peak at 284.6 eV.[3-5] The chemical composition of the samples was measured by the inductively coupled plasma optical emission spectroscopy (ICP-OES) on Agilent ICP-OES730.

Electrochemical measurements

All electrochemical measurements were carried out on a CHI 760E electrochemical workstation (Shanghai Chenhua Instrument Co., Ltd., China) within a three-electrode cell in 0.5 M H_2SO_4 at room temperature. A glassy carbon electrode (GCE) with a diameter of 3 mm covered by a thin catalyst film, a graphite rod and Ag/AgCl electrode (saturated KCl-filled) were used as the working, counter electrode and reference electrodes, respectively. All potentials were referenced to the reversible hydrogen electrode (RHE) by the equation: E (vs. RHE) = E (vs. Ag/AgCl) + 0.197 + 0.059 × pH. To prepare the catalyst-covered working electrode, a droplet of precursor solution (5 μl) was drop-cast onto the surface of the polished GCE by a micro pipette (the loading for all catalysts was fixed at 0.283 mg cm^{-2}), followed by plasma treatment at room temperature. Afterward, Nafion solution (5 μl, 0.05 wt.%) was added onto the surface of the catalyst to ensure firm attachment during electrochemical measurements. Before starting the electrochemical

experiments, the electrolyte was de-aerated by purging with N_2 for 30 min. Linear sweep voltammetry (LSV) curves were recorded at a scan rate of 5 mV s^{-1}. All polarization curves were corrected with iR-compensation. The double-layer capacitance was estimated by conducting cyclic voltammetry (CV) at various scan rates of 10, 20, 30, 40, and 50 mV s^{-1} between 0.1 and 0.2 V (vs. RHE). We calculated the electrochemical surface area (ECSA) using the formula $S_{\mathrm{ECSA}} = \frac{C_{\mathrm{dl}} \times S_{\mathrm{GEO}}}{\mathrm{SC}}$.[8] C_{dl} is the double-layer capacitance in Figure 2e and S_{GEO} is the geometric area of the working electrode (0.0707 cm^2 for the glassy carbon electrode with a diameter of 3 mm). SC is the specific capacitance of a smooth flat surface (1 cm^2) under identical electrolyte conditions. For our estimates of ECSA, a general value of 29 μF cm^{-2} for SC, as reported in prior work, was used.[9] Electrochemical impedance spectroscopy (EIS) measurements were performed at a potential of -0.25 V (vs. RHE) over a frequency range from 0.01 Hz to 100 kHz with a 5 mV sinusoidal perturbation. CV was conducted at a scan rate of 50 mV s^{-1} for 2000 cycles in the potential range of 0.1 to -0.4 V (vs. RHE) to test the cycling stability. The long-term stability was examined by recording a chronoamperometric curve at a constant overpotential of 400 mV (vs. RHE) for 24 h without iR-compensation. For the chronoamperometry test, Pt mesh was used as the counter electrode due to the instability of graphite rod under high currents. In order to avert possible platinum contamination during measurements, cathode and anode compartments were separated using Nafion 117 membrane in H-type electrochemical cell. ZView software was used to fit the EIS Nyquist plots. For whole cell water electrolysis, carbon paper (CP, CeTech, Taiwan) was cut into 1 × 1 cm^2 pieces and used as the catalyst support. For cathode, 250 μl of the precursor suspension (4 mg mL^{-1}, $(NH_4)_2MoS_4/(NH_4)_2WS_4$=1:1) was loaded onto a piece of CP, followed by the identical plasma treatment as described above. For comparison, 10 mg of 20 wt.% Pt/C (Tanaka, Tokyo, Japan) and 100 μl of 5 wt.% Nafion solution were dispersed in 900 μl of 7:2(v/v) water/ethanol with 30 min bath-sonication to form a Pt/C ink. 100 μl of the Pt/C ink was loaded onto a piece of CP. For anode, 10 mg of RuO_2 (99.9%, Sigma Aldrich, St. Louis, USA) and 100 μl of 5 wt.% Nafion solution were dispersed in 900 μl of 7:2(v/v) water/ethanol with 30 min bath-sonication to form a RuO_2 ink. 100 μl of the RuO_2 ink was loaded onto a piece of CP.

Computational methods

All DFT calculations were implemented with ORCA package,[10] an advanced first principles

calculation software. All calculations were implemented with lanl2dz double-zeta basis set [11-13] with effective core potentials in W and Mo, taking dispersion correction into account with Grimme's DFT-D3.[14] All calculations were employed with GGA-PBE functional,[11] which is low-cost and accurate for transition metal elements.[16] Firstly, the spin multiplicity test was carried out, in order to find the correct configuration of clusters in the ground state, the lowest energy state was selected from all optimized structures with different spin multiplicity, showing no single electron in our models. Then, analytical frequency calculations were performed to obtain thermodynamic data. Mayer bond order and electronic localization function were generated with Multiwfn.[17]

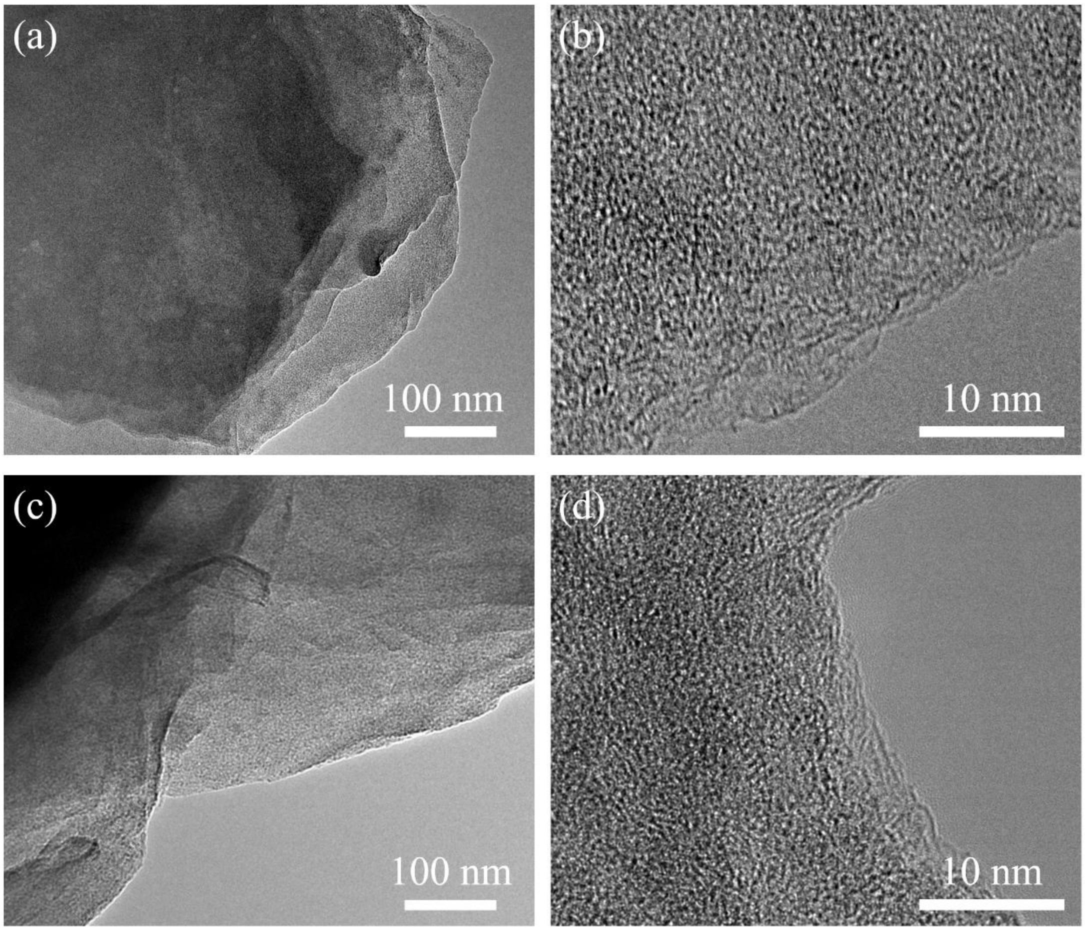

Figure S1 The medium and high-resolution TEM images of (a, b) a-$MoWS_x$/RGO@1:2-10 min and (c, d) a-$MoWS_x$/RGO@2:1-10 min.

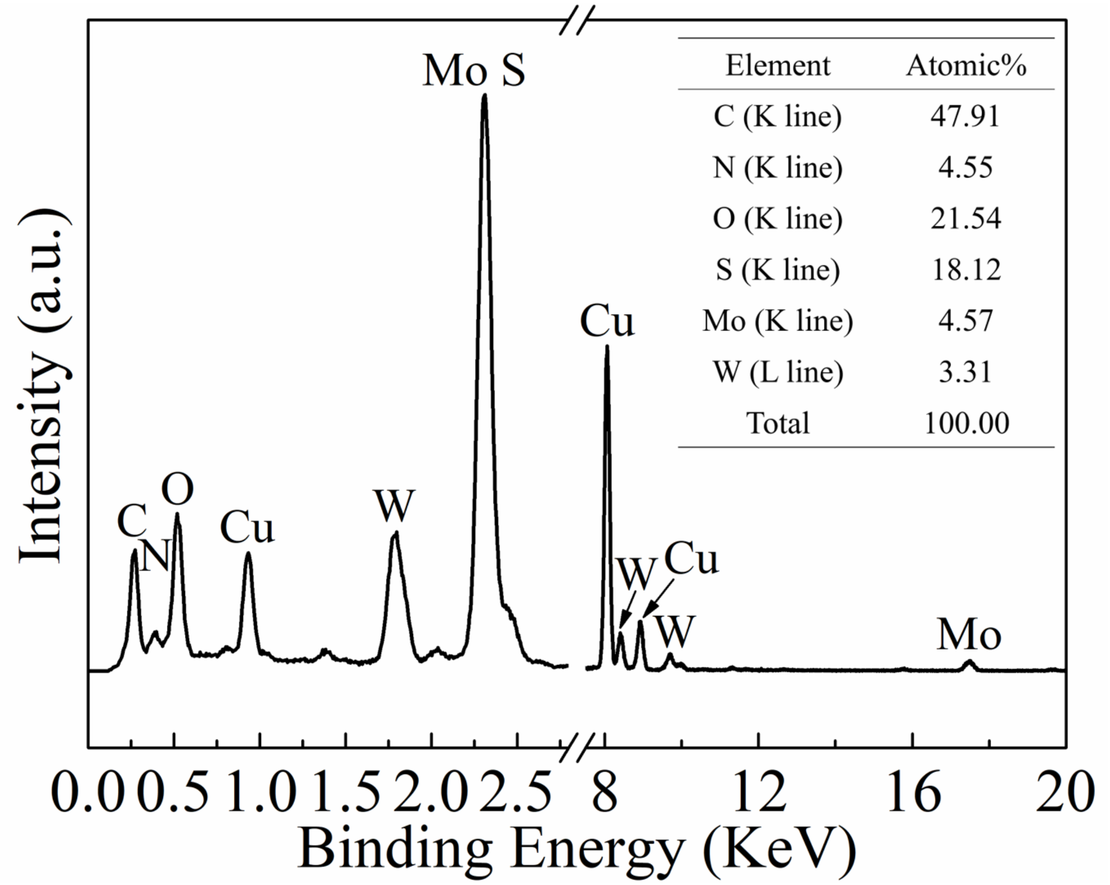

Figure S2 The EDX spectrum of a-$MoWS_x$/RGO@1:1-10 min.

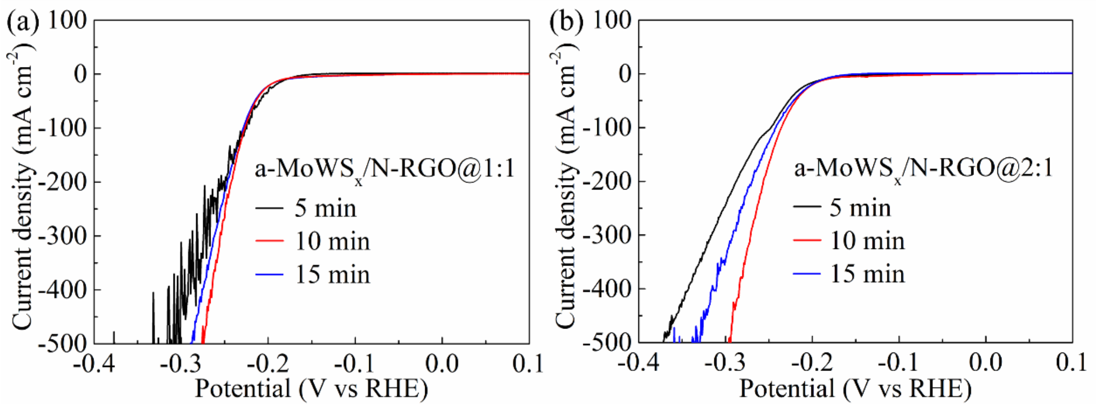

Figure S3 (a) LSV polarization curves for a-$MoWS_x$/N-RGO@1:1 with varied plasma treatment time. (b) LSV polarization curves for a-$MoWS_x$/N-RGO@2:1 with varied plasma treatment time.

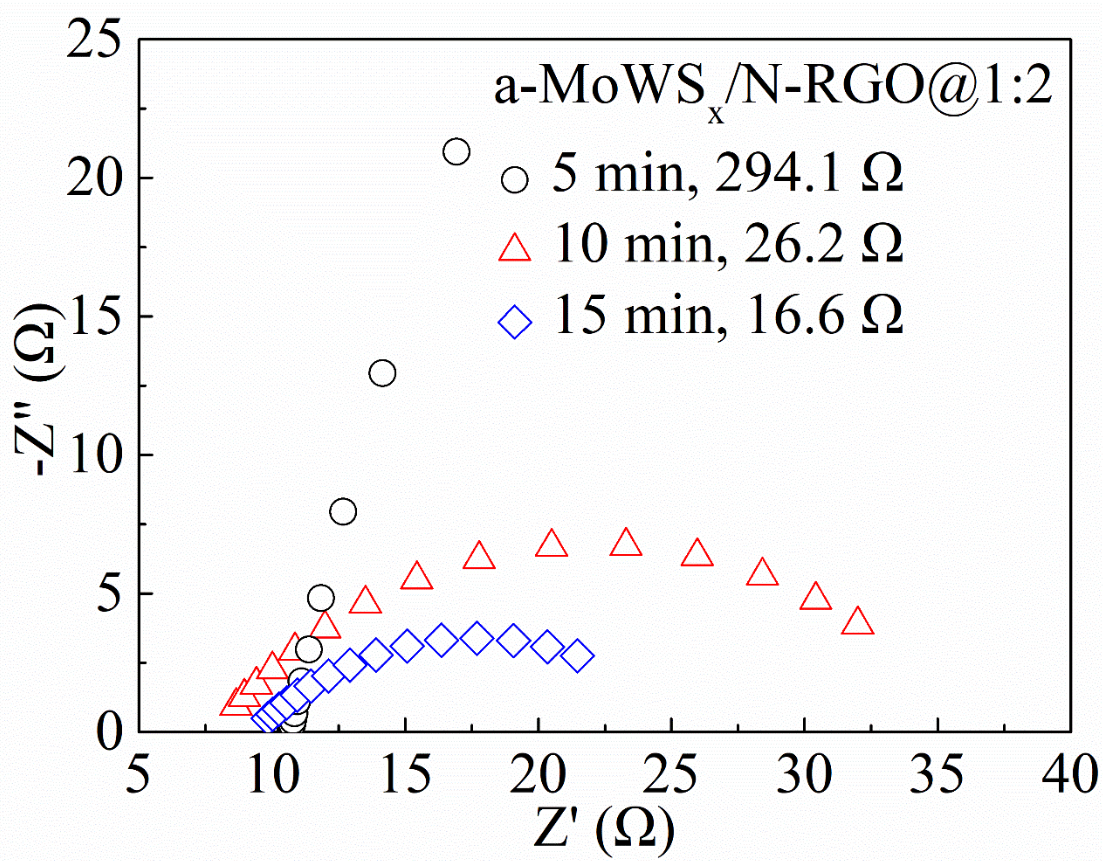

Figure S4 Nyquist plots of the electrochemical impedance spectra of a-$MoWS_x$/N-RGO@1:2 with varied plasma treatment duration.

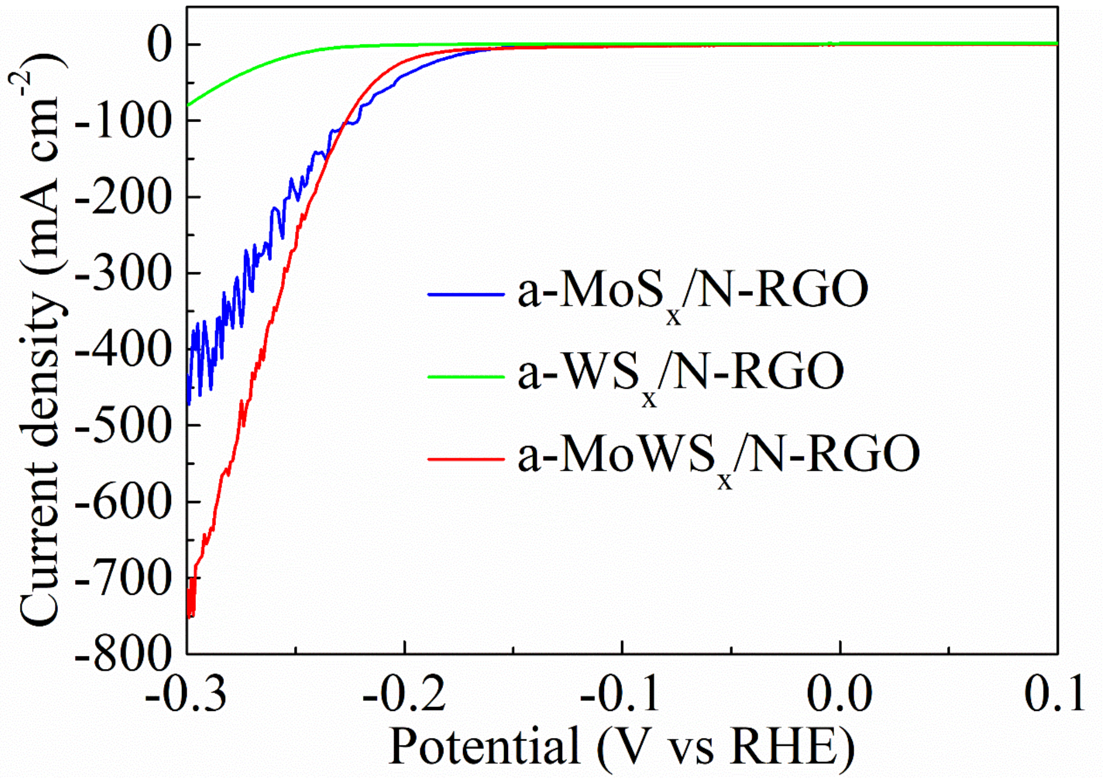

Figure S5 LSV polarization curves of a-MoS$_x$/N-RGO, a-WS$_x$/N-RGO and a-MoWS$_x$/N-RGO in 0.5 M H_2SO_4.

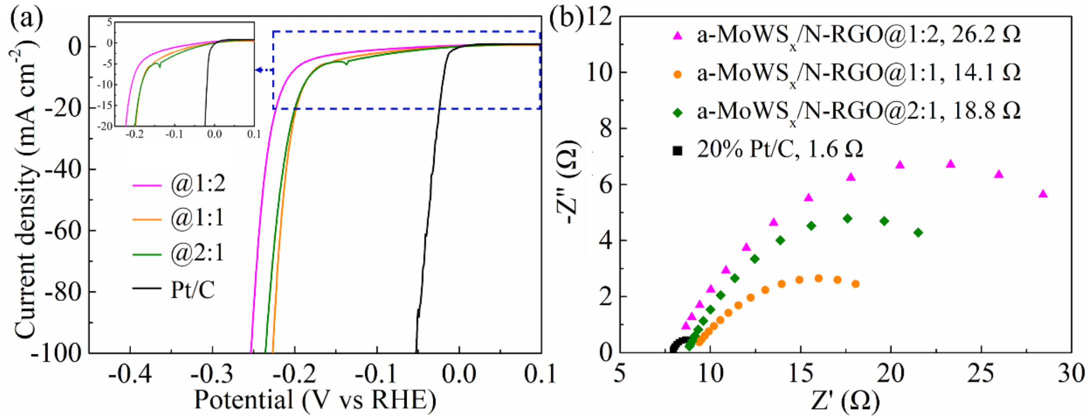

Figure S6 (a) Enlarged view of low overpotential region in Fig 2c. (b) Nyquist plots of the electrochemical impedance spectra. The onset potential of a-$MoWS_x$/N-RGO@1:2, a-$MoWS_x$/N-RGO@1:1, a-$MoWS_x$/N-RGO@2:1 and Pt/C were 96 mV, 62 mV, 46 mV and 8mV, respectively.

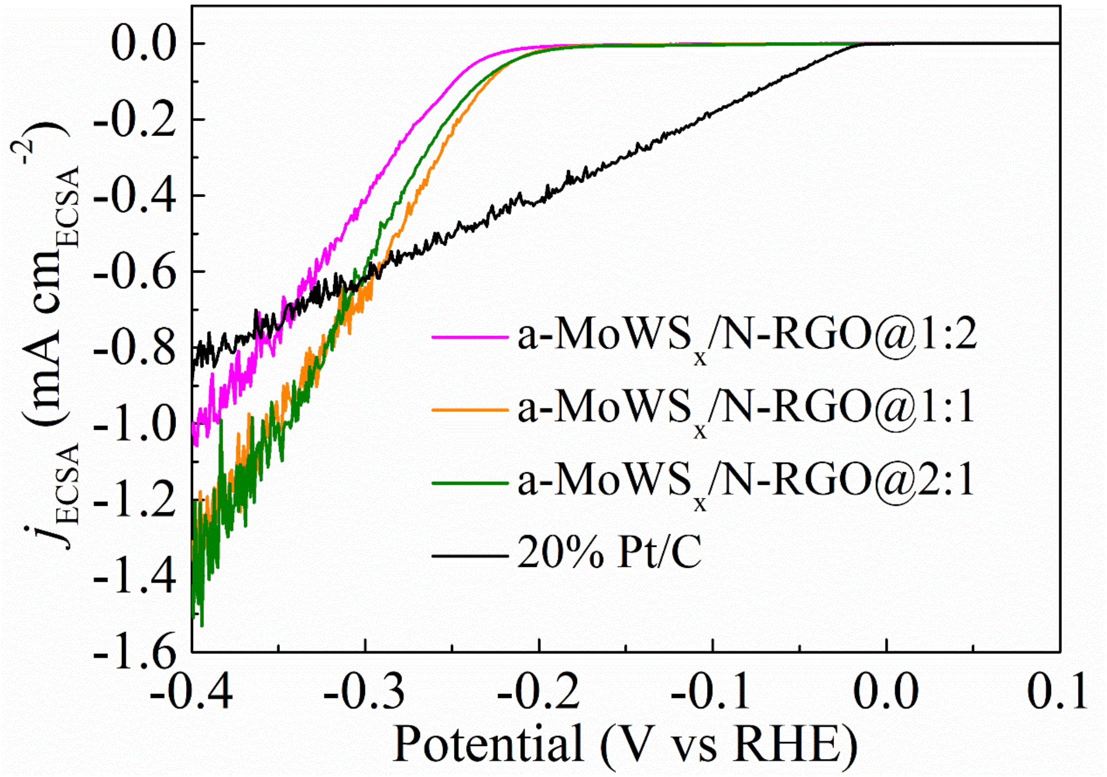

Figure S7 Polarization curves from Figure 2c normalized to the electrochemical surface area (ECSA).

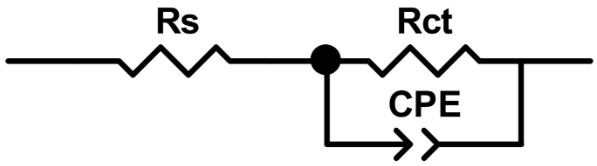

Figure S8 The equivalent circuit model for EIS response fitting. The equivalent circuit consists of a solution resistance (R_s), in series connection with a parallel unit of a constant phase element (CPE) and a charge-transfer resistance (R_{ct}).

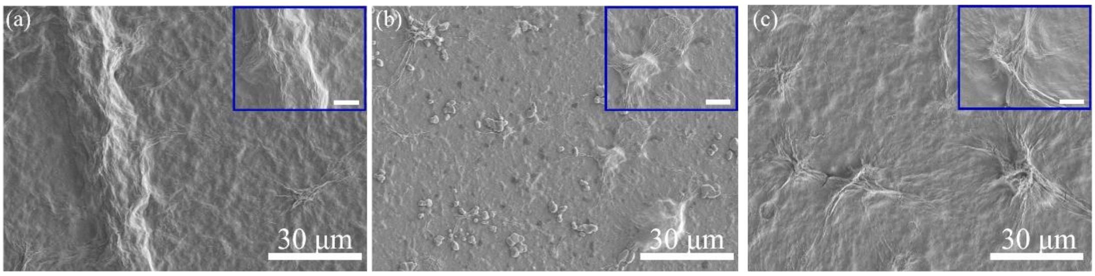

Figure S9 SEM images of (a) a-$MoWS_x$/N-RGO@1:2, (b) a-$MoWS_x$/N-RGO@1:1, (c) a-$MoWS_x$/N-RGO@2:1. The insets in f-h are local magnification images displaying the morphologies of wrinkles and humps (the scale bar is 6 μm).

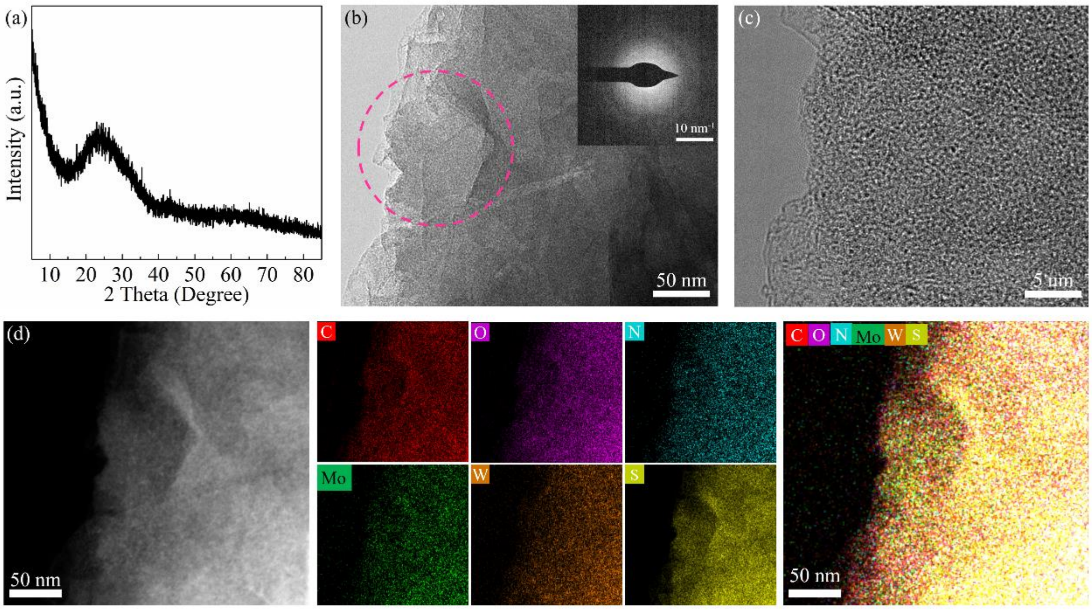

Figure S10 Structural, morphological and elemental characterization of the catalyst after the long-term durability test. (a) XRD pattern, (b) medium-magnification TEM image (inset: the SAED pattern of the area marked by the pink dotted circle), (c) high-resolution TEM image and (d) HAADF-STEM image and corresponding elemental mapping of a-$MoWS_x$/N-RGO@1:1-10 min after 12 hours of CA test.

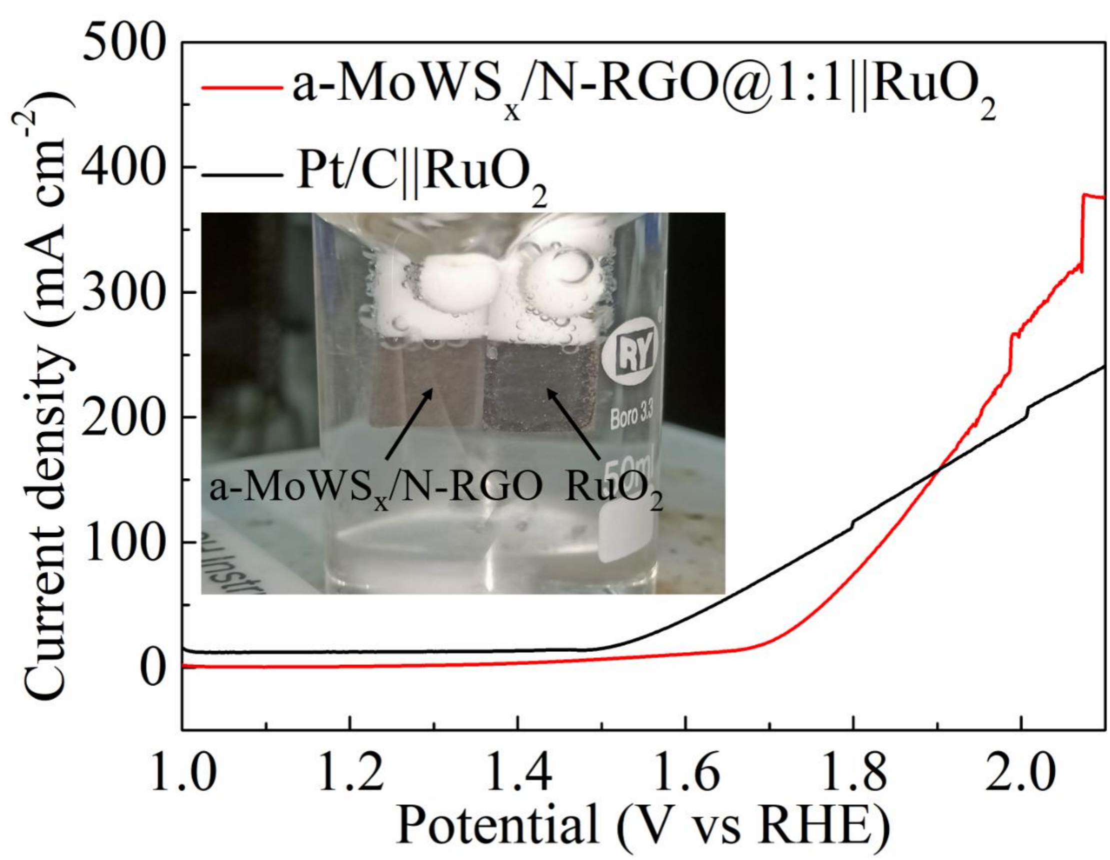

Figure S11 LSV comparing the water-splitting performance of a-$MoWS_x$/N-RGO@1:1-RuO_2 couple and Pt/C-RuO_2 couple (inset: the photograph for the whole cell water electrolyzer).

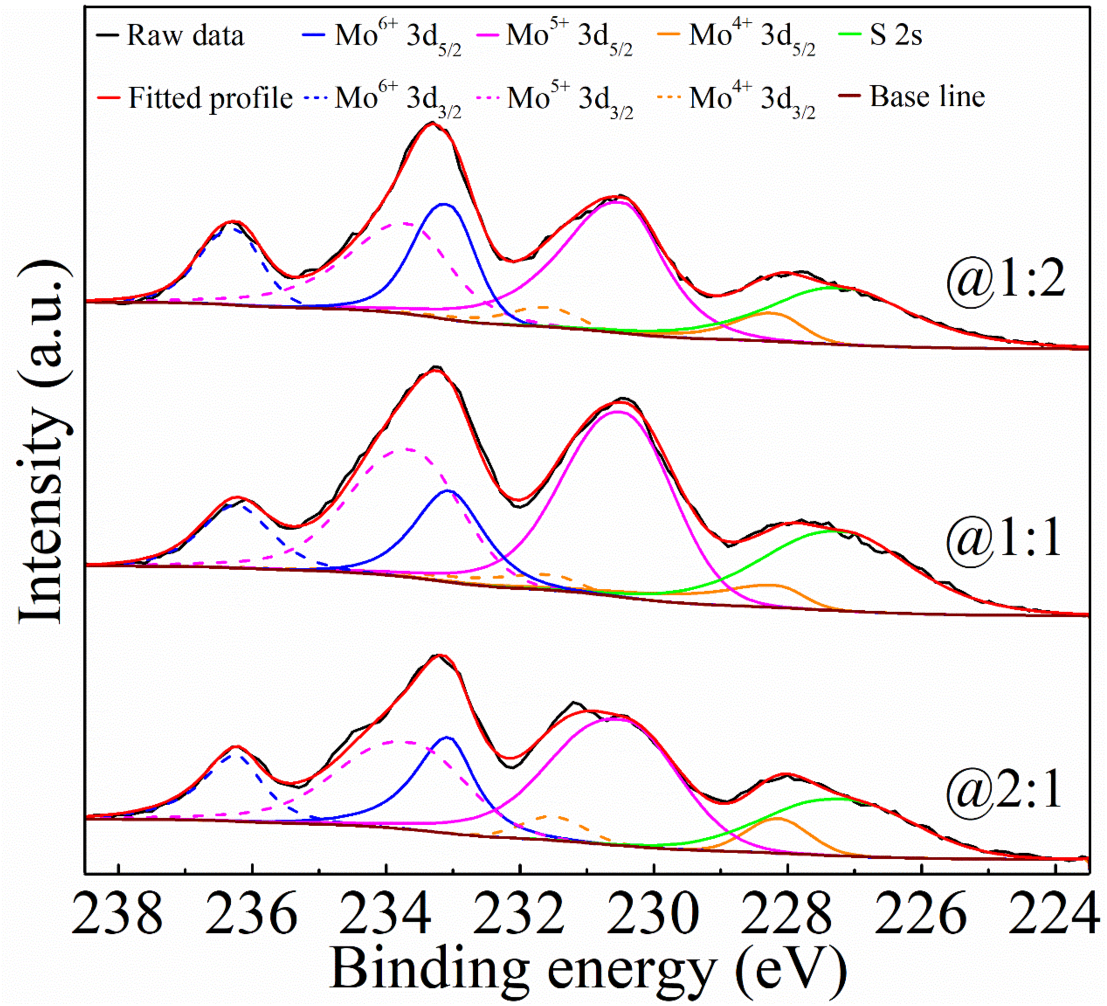

Figure S12 High-resolution Mo 3d XPS spectra of a-$MoWS_x$/N-RGO with varied precursor ratio and a fixed plasma treatment time of 10 min.

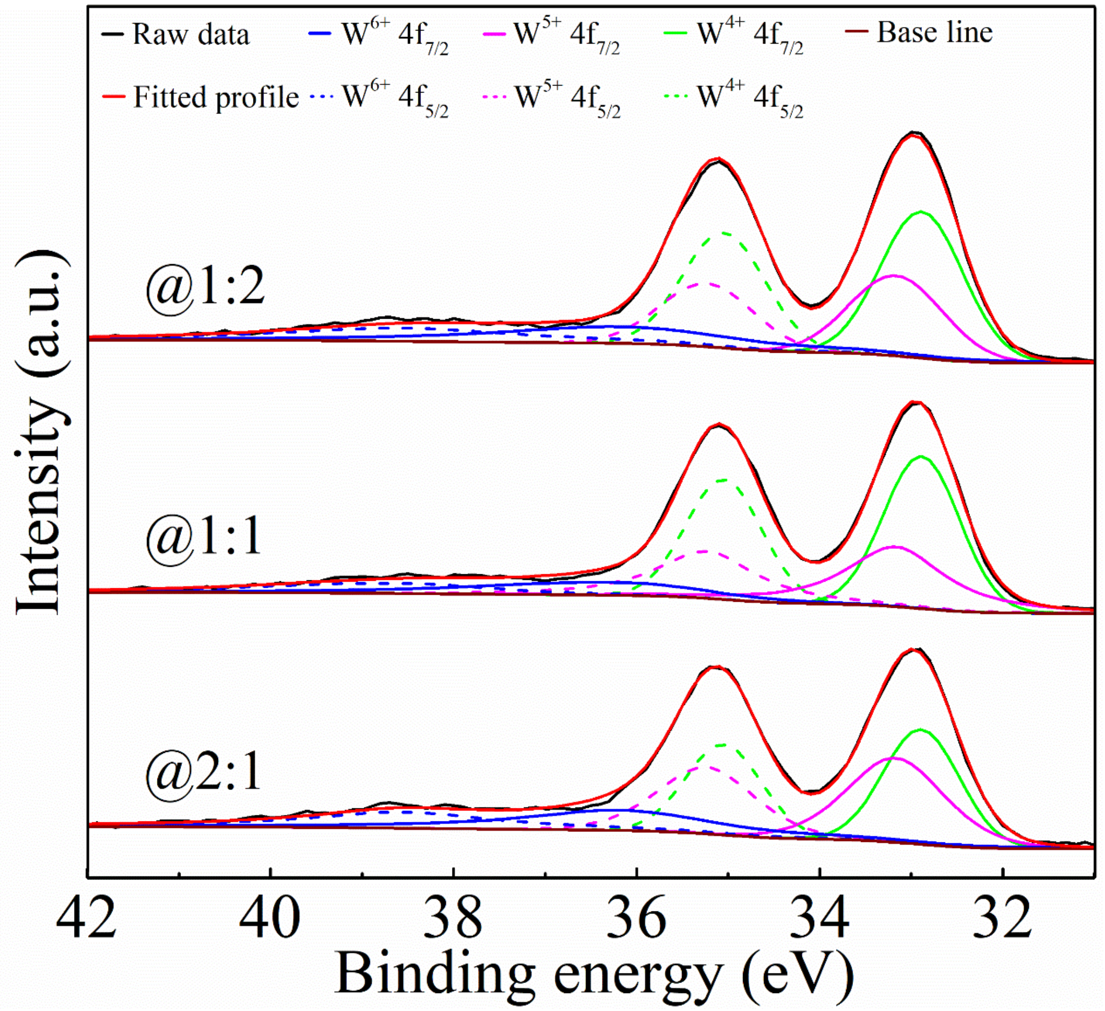

Figure S13 High-resolution W 4f XPS spectra of a-$MoWS_x$/N-RGO with varied precursor ratio and a fixed plasma treatment time of 10 min.

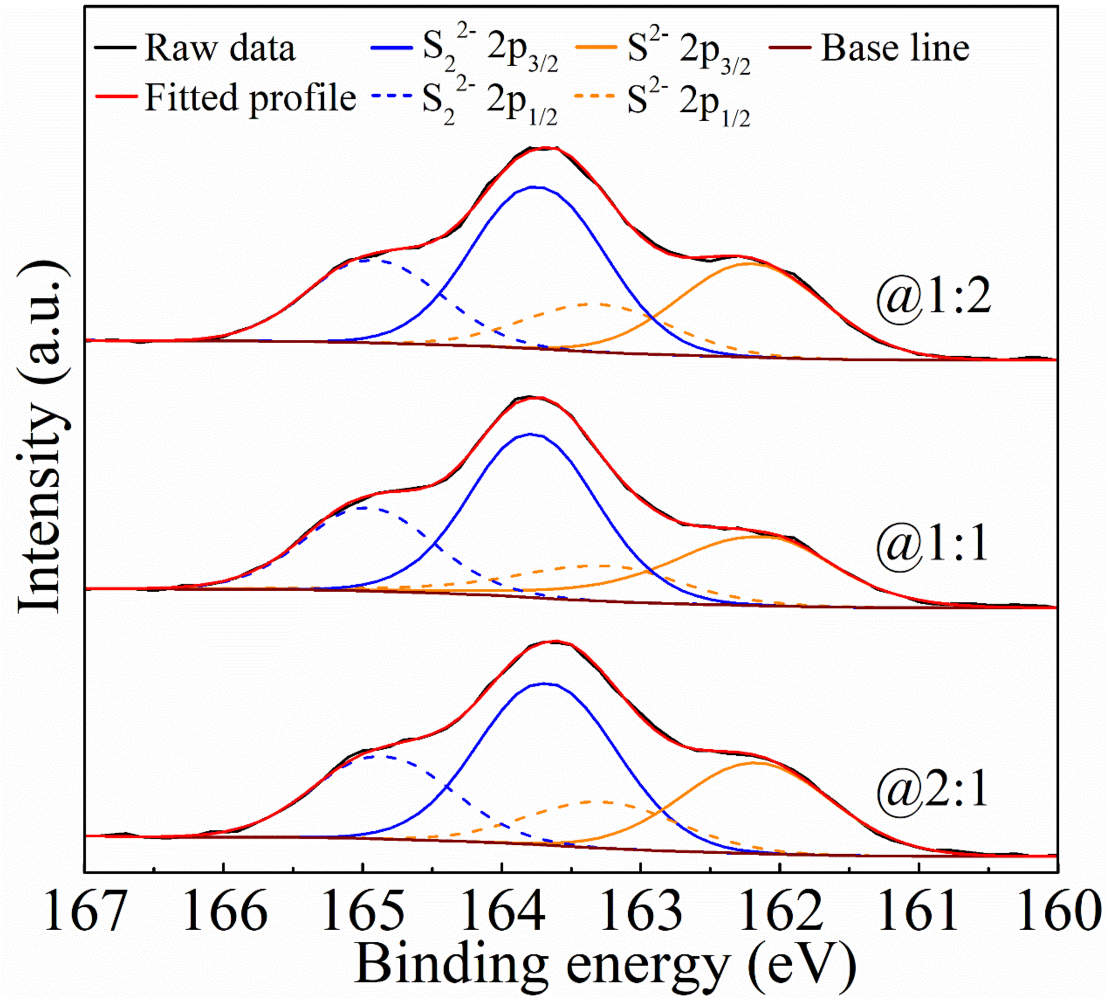

Figure S14 High-resolution S 2p XPS spectra of a-MoWS$_x$/N-RGO with varied precursor ratio and a fixed plasma treatment time of 10 min.

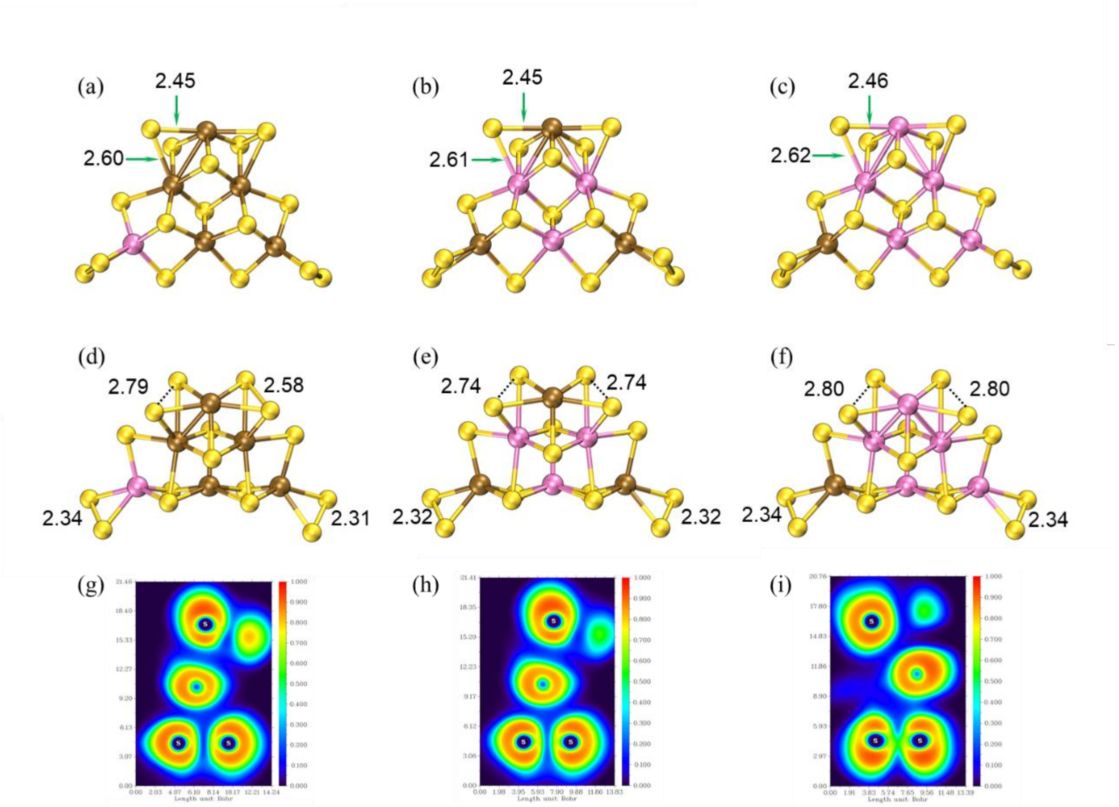

Figure S15 Three specific cluster models with different W-content at a random site (mauve atom represents W element.), with Mo_5WS_{16} (a) and (d), $Mo_3W_3S_{16}$ (b) and (e), and MoW_5S_{16} (c) and (f) in different visual angle. The number in the figure indicates the distance of S-metal or S-S. Pseudo-color graphs reflect electronic localization function with three typical catalytic S-sites in Mo_5WS_{16}, with (g) double S^{2-}, (h) double S^{2-} despite a shorter distance of S-S (2.58 Angstrom) which is not enough to make S-atom interact each other, and (i) terminal S_2^{2-} whose S-S distance reaches about 2.32±0.02 Angstrom, making electron partially localized in S-S bond which indicates bond formation of S_2^{2-}.

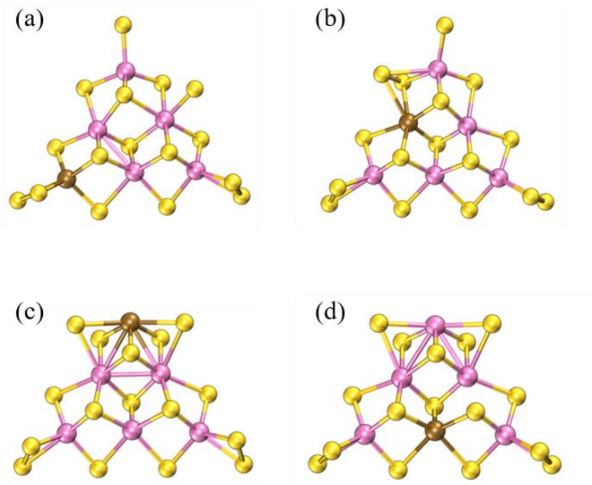

Figure S16 All possible structures of $[MoW_5S_{16}]^{2-}$ with unstable geometry (a) and (b), and stable geometry (c) and (d).

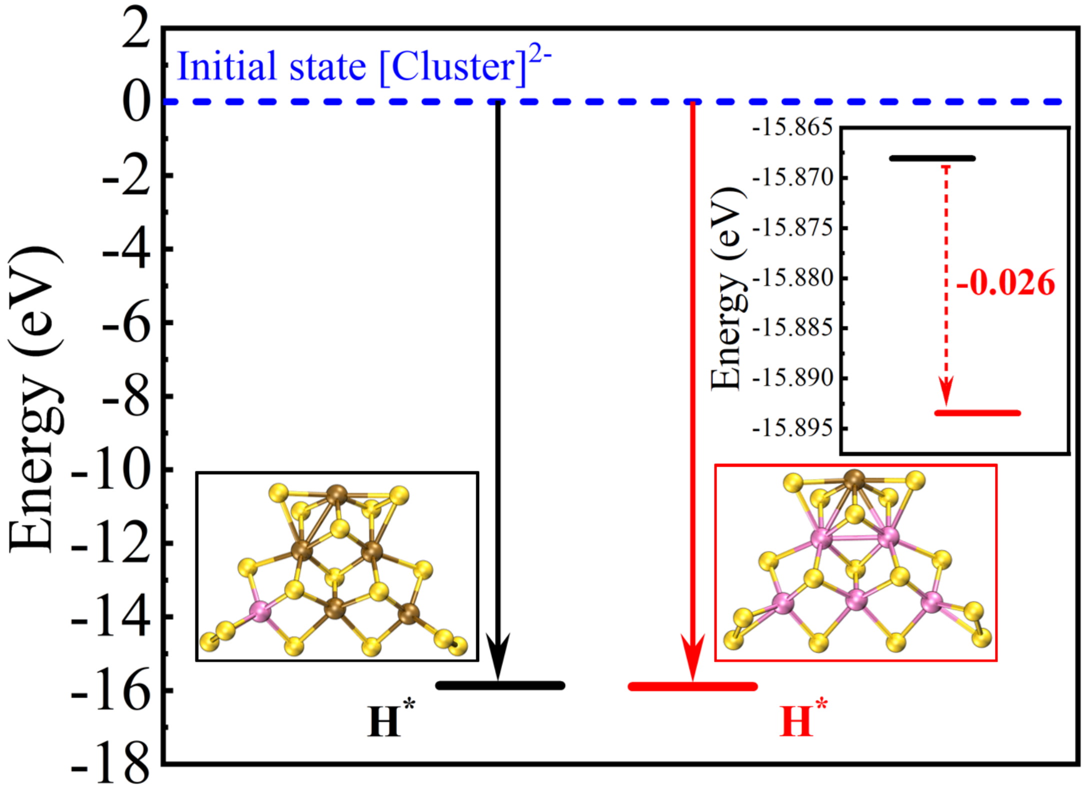

Figure S17 Electronic energy difference between $[Mo_5WS_{16}]^{2-}$ (black) and $[MoW_5S_{16}]^{2-}$ (red).

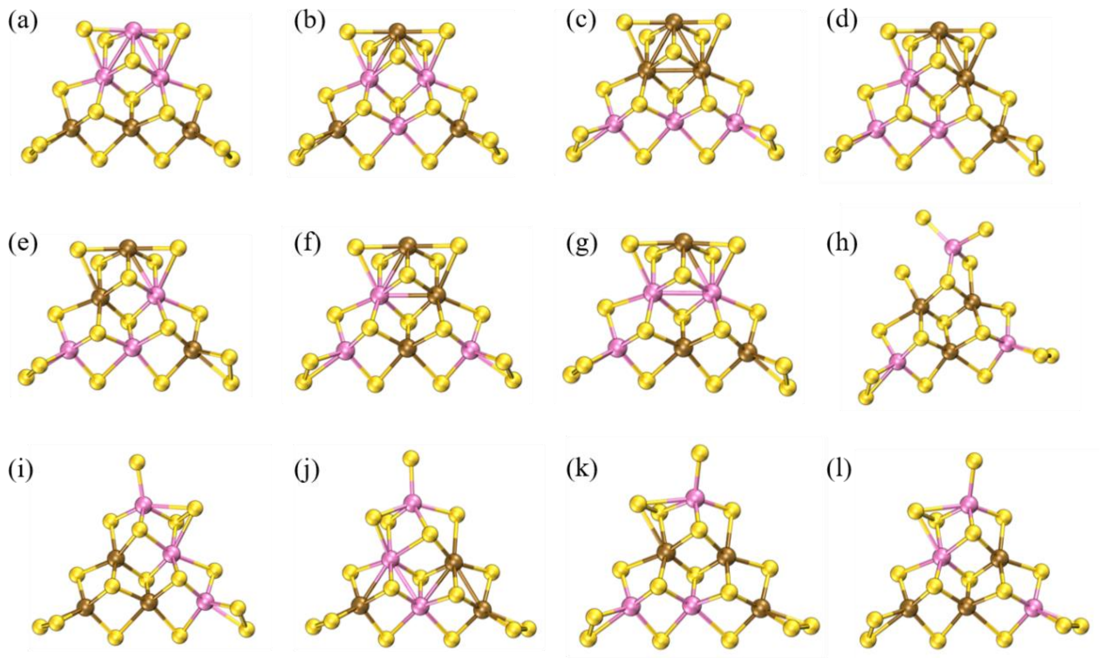

Figure S18 All possible structures of $[Mo_3W_3S_{16}]^{2-}$ with stable geometry (a) - (g), and unstable geometry (h) - (l).

Table S1. Comparison of the performances of MoS_2-based catalysts toward high-current-density HER in 0.5 M H_2SO_4.

Catalyst	Counter electrode	Overpotential at 1000 mA cm^{-2} (mV)	Tafel slope (mV dec^{-1})	Catalyst loading (mg cm^{-2})	Reference
a-$MoWS_x$/N-RGO	graphite rod	348	41	0.283	This work
MoS_2/CNF-PHH-U [1]	graphite rod	450	69	N/A	[18]
Superaerophobic MoS_2/Ti	graphite rod	>400	51	0.12	[19]
Co/Se-MoS_2-NF [2]	graphite rod	382	67	0.5	[20]
NP-MoS_2/CC [3]	graphite rod	~480	58.4	1.2	[21]
MoS_2/Mo_2C	graphite rod	412	60	10	[22]
MoS_2/Mo_2C heterostructure	graphite rod	227	43	0.3	[23]

[1] Edge-exposed MoS_2/carbon nanofiber

[2] Co/Se-codoped MoS_2 nanofoam

[3] N and P codoped MoS_2 array anchored on carbon cloth

The catalysts in orange background are heteroatom-doped MoS_2-based catalysts. The catalysts in blue background are MoS_2-based heterostructure catalysts.

Table S2. ICP-OES results for a-$MoWS_x$/N-RGO@1:1-10 min before and after the stability test.

	before the stability test (wt%)	after the stability test (wt%)
Mo	15.18	15.20
S	20.62	20.92
W	25.87	25.29

Table S3. The deconvolution results of core-level S 2p spectra of the final samples with varied precursor ratio.

Precursor ratio	S_2^{2-} (%)	S^{2-} (%)
@1:2	61.4	38.6
@1:1	66.4	33.6
@2:1	63.0	37.0

Table S4. The atomic content of Mo, W and S in the final samples with varied precursor ratio.

	Mo (at.%)	W (at.%)	S (at.%)	Mo:W (experiment)	Mo:W (DFT model)
@1:2	3.13	5.17	15.48	1:1.65	1:5
@1:1	4.23	3.76	17.20	1.13:1	1:1
@2:1	6.24	2.87	23.19	2.17:1	5:1

Table S5. Comparison of Gibbs free energies of a-$MoWS_x$ and Pt crystal.

	ΔG_{H^*}		
	X	$[X]^-$	$[X]^{2-}$
Site 2 in Figure 3b	-0.057	-0.091	0.120
Pt	-0.022	-0.279	-0.302

X denotes Mo_5WS_{16} in Figure 3b or Pt.

References

[1]. Zhang, D.; Wang, F.; Fan, X.; Zhao, W.; Cui, M.; Li, X.; Liang, R.; Ou, Q.; Zhang, S. *Carbon* **2022,** 187, 386-395.

[2]. Zhou, H.; Liu, Y.; Zhang, L.; Li, H.; Liu, H.; Li, W. *J. Colloid Interface Sci.* **2019,** 533, 287-296.

[3]. Zou, X.; Zhang, Y. *Chem. Soc. Rev.* **2015,** 44, (15), 5148-5180.

[4]. Yang, C.; Gong, J.; Zeng, P.; Yang, X.; Liang, R.; Ou, Q.; Zhang, S. *Appl. Surf. Sci.* **2018,** 452, 481-486.

[5]. Palchan, I.; Crespin, M.; Estrade-Szwarckopf, H.; Rousseau, B. *Chem. Phys. Lett.* **1989,** 157, (4), 321-327.

[6]. Lee, B.; Park, S.-Y.; Kim, H.-C.; Cho, K.; Vogel, E. M.; Kim, M. J.; Wallace, R. M.; Kim, J. *Appl. Phys. Lett.* **2008,** 92, (20).

[7]. Chang, Y.-H.; Wu, F.-Y.; Chen, T.-Y.; Hsu, C.-L.; Chen, C.-H.; Wiryo, F.; Wei, K.-H.; Chiang, C.-Y.; Li, L.-J. *Small* **2014,** 10, (5), 895-900.

[8]. Chen, N.; Zhang, W.; Zeng, J.; He, L.; Li, D.; Gao, Q. *Appl. Catal., B* **2020,** 268, 118441.

[9]. Zheng, X.; De Luna, P.; García de Arquer, F. P.; Zhang, B.; Becknell, N.; Ross, M. B.; Li, Y.; Banis, M. N.; Li, Y.; Liu, M.; Voznyy, O.; Dinh, C. T.; Zhuang, T.; Stadler, P.; Cui, Y.; Du, X.; Yang, P.; Sargent, E. H. *Joule* **2017,** 1, (4), 794-805.

[10]. Neese, F. *WIREs Comput. Mol. Sci.* **2022,** e1606.

[11]. Hay, P. J.; Wadt, W. R. *J. Chem. Phys.* **1985,** 82, (1), 299-310.

[12]. Hay, P. J.; Wadt, W. R. *J. Chem. Phys.* **1985,** 82, (1), 270-283.

[13]. Wadt, W. R.; Hay, P. J. *J. Chem. Phys.* **1985,** 82, (1), 284-298.

[14]. Grimme, S.; Antony, J.; Ehrlich, S.; Krieg, H. *J. Chem. Phys.* **2010,** 132, (15), 154104.

[15]. Perdew, J. P.; Burke, K.; Ernzerhof, M. *Phys. Rev. Lett.* **1996,** 77, (18), 3865-3868.

[16]. Chan, B.; Gill, P. M. W.; Kimura, M. *J. Chem. Theory Comput.* **2019,** 15, (6), 3610-3622.

[17]. Lu, T.; Chen, F. *J. Comput. Chem.* **2012,** 33, (5), 580-592.

[18]. Zhang, Z.; Wang, Y.; Leng, X.; Crespi, V. H.; Kang, F.; Lv, R. *ACS Appl. Energy Mater.* **2018,** 1, (3), 1268-1275.

[19].Lu, Z.; Zhu, W.; Yu, X.; Zhang, H.; Li, Y.; Sun, X.; Wang, X.; Wang, H.; Wang, J.; Luo, J.; Lei, X.; Jiang, L. *Adv. Mater.* **2014,** 26, (17), 2683-2687.

[20]. Zheng, Z.; Yu, L.; Gao, M.; Chen, X.; Zhou, W.; Ma, C.; Wu, L.; Zhu, J.; Meng, X.; Hu, J.; Tu, Y.; Wu, S.; Mao, J.; Tian, Z.; Deng, D. *Nat. Commun.* **2020,** 11, (1), 3315.

[21]. Sun, K.; Zeng, L.; Liu, S.; Zhao, L.; Zhu, H.; Zhao, J.; Liu, Z.; Cao, D.; Hou, Y.; Liu, Y.; Pan, Y.; Liu, C. *Nano Energy* **2019,** 58, 862-869.

[22]. Zhang, C.; Luo, Y.; Tan, J.; Yu, Q.; Yang, F.; Zhang, Z.; Yang, L.; Cheng, H.-M.; Liu, B. *Nat. Commun.* **2020,** 11 (1), 3724.

[23]. Luo, Y.; Tang, L.; Khan, U.; Yu, Q.; Cheng, H.-M.; Zou, X.; Liu, B. *Nat. Commun.* **2019,** 10, (1), 269.
